# Population encoding of stimulus features along the visual hierarchy

**DOI:** 10.1073/pnas.2317773121

**Published:** 2024-01-16

**Authors:** Luciano Dyballa, Andra M. Rudzite, Mahmood S. Hoseini, Mishek Thapa, Michael P. Stryker, Greg D. Field, Steven W. Zucker

**Affiliations:** ^a^Department of Computer Science, Yale University, New Haven, CT 06511; ^b^Department of Neurobiology, Duke University, Durham, NC 27708; ^c^Department of Physiology, University of California, San Francisco, CA 94143; ^d^Department of Ophthalmology, David Geffen School of Medicine, Stein Eye Institute, University of California, Los Angeles, CA 90095; ^e^Kavli Institute for Fundamental Neuroscience, University of California, San Francisco, CA 94143; ^f^Department of Biomedical Engineering, Yale University, New Haven, CT 06511

**Keywords:** computational neuroscience, encoding manifold, retina, visual cortex, deep networks

## Abstract

The retina and primary visual cortex (V1) both exhibit diverse neural populations sensitive to diverse visual features. Yet it remains unclear how neural populations in each area partition stimulus space to span these features. One possibility is that neural populations are organized into discrete groups of neurons, with each group signaling a particular constellation of features. Alternatively, neurons could be continuously distributed across feature-encoding space. To distinguish these possibilities, we presented a battery of visual stimuli to the mouse retina and V1 while measuring neural responses with multi-electrode arrays. Using machine learning approaches, we developed a manifold embedding technique that captures how neural populations partition feature space and how visual responses correlate with physiological and anatomical properties of individual neurons. We show that retinal populations discretely encode features, while V1 populations provide a more continuous representation. Applying the same analysis approach to convolutional neural networks that model visual processing, we demonstrate that they partition features much more similarly to the retina, indicating they are more like big retinas than little brains.

## Significance

We present a method for understanding the functional relationships among large populations of neurons in complex circuits such as the retina and primary visual cortex that allows a global comparison with artificial neural networks. We first show that the mouse retina and visual cortex encode visual scenes in fundamentally different ways: Populations of neurons in the retina discretely sample visual features, while neurons in the primary visual cortex provide a continuous representation. This settles, for the mouse at least, a classical debate about the role of channels in neural information processing. We then demonstrate that two popular convolutional neural networks fail to reproduce fundamental features of real cortical populations.

The output of the mouse retina is formed by a set of about 40 distinct types of retinal ganglion cells (RGCs) (1, 2). These RGC types exhibit distinct morphologies, gene expression profiles, and visual responses. This has generated a coherent perspective on retinal output: Distinct cell types signal distinct visual features to downstream brain areas. How are these distinct features organized in the cortex? Many studies have focused on parallel pathways in sensory systems and in the visual system in particular (3, 4). One possibility is that the visual features signaled by parallel pathways originating from the different RGC classes would be combined to produce new groupings of features in the cortex (5, 6). An alternative possibility is that visual cortical organization is a continuum, fundamentally different from that of the retina, despite the mounting evidence for distinct neuronal identities in V1, with cortical cell types of different morphologies, transcriptomes, and intrinsic physiological properties (7).

To determine whether stimulus features are represented similarly over the parallel pathways between the retina and visual cortex, we have devised the “neural encoding manifold”—an analysis of the organization of neurons signaling a wide range of visual response features. We have applied it to both retina and V1 using matched stimulus ensembles across the two populations of neurons. Our conclusion is that the population-level representations of stimulus features are fundamentally different between the retina and visual cortex, the former organized as distinct clusters while the latter as a continuum.

## An Ensemble of Grating and Flow Stimuli.

**Fig. 1.**
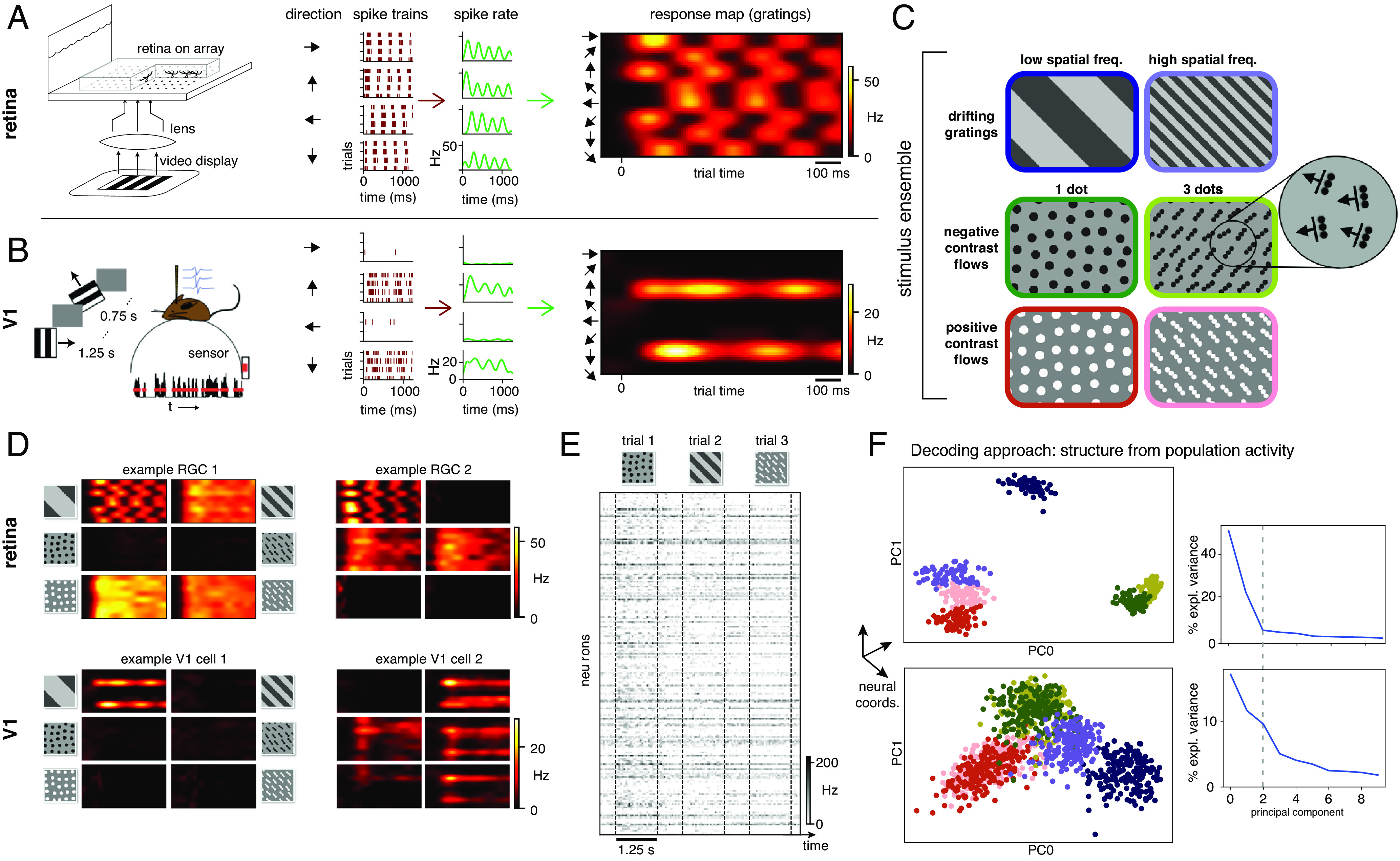
Experimental set-up and stimulus ensemble. (*A* and *B*) MEA measurements from mouse RGCs and V1 neurons. Spikes from drifting stimuli are trial-averaged and collected into response maps. Examples shown from an ON-center RGC (*A*) and an orientation-selective cell in V1 (*B*). (*C*) The stimulus ensemble consists of low and high spatial frequency gratings and positive or negative contrast flow patterns composed of either single dots or 3-dot oriented line segments moving in eight directions separated by 45° (*Materials and Methods*). The illustrated 3×2 arrangement will be used throughout. (*D*) Two example RGC (*Top*) and V1 (*Bottom*) response profiles illustrating the diverse response dynamics to the different stimuli. Left cells identical to (*A* and *B*). (*E*) Rasters showing population responses: neurons × spike trains. (*F*) *Left:* Example of standard embedding approaches (9–12) arranges trials in neural coordinates and enables “decoding,” i.e., inferring the stimulus from the neural state (top is retina, bottom is V1). Each point represents the response of the neural population to a given stimulus on a trial in a low-dimensional space determined by principal components analysis (*Materials and Methods*); colors indicate the different stimuli. *Right:* Principal value spectrum associated with the principal components for RGCs (*Top*) and V1 (*Bottom*): Although stimulus decoding is clear in both, the cortex appears to require more dimensions to capture variability in the responses.

To identify the degree of similarity between population-level neural representations of stimuli in the retina and V1, we performed multi-electrode array (MEA) recordings in both areas utilizing matched stimuli and analysis procedures. MEAs were used to measure the responses of mouse RGCs ex vivo, and responses of mouse V1 neurons in vivo (Fig. 1 *A* and *B*). Sinusoidal drifting gratings and optical flow stimuli were presented at matched spatial frequencies and contrasts in the retina and V1 experiments (Fig. 1*C*). Drifting gratings were presented at two spatial frequencies and drifted in eight directions. Flow stimuli were presented at two contrasts (positive and negative), consisting of dots and oriented line segments, and drifted in eight directions. Flow stimuli were chosen because they mimic certain features of naturalistic stimuli, and previous work has shown that they engage nonlinearities in V1 that are not predicted based on the responses to gratings (8). In addition, flow and grating stimuli were spatially isotropic (or repeating), allowing us to study response properties independent of receptive field location. Individual neuronal responses were summarized by a two-dimensional response map, where each row shows the response to a particular stimulus direction of motion; one such map was produced for each neuron and for each of the six stimuli presented (Fig. 1*D*).

## Organizing Neurons, Stimuli, and Responses via the Encoding Manifold

**Fig. 2.**
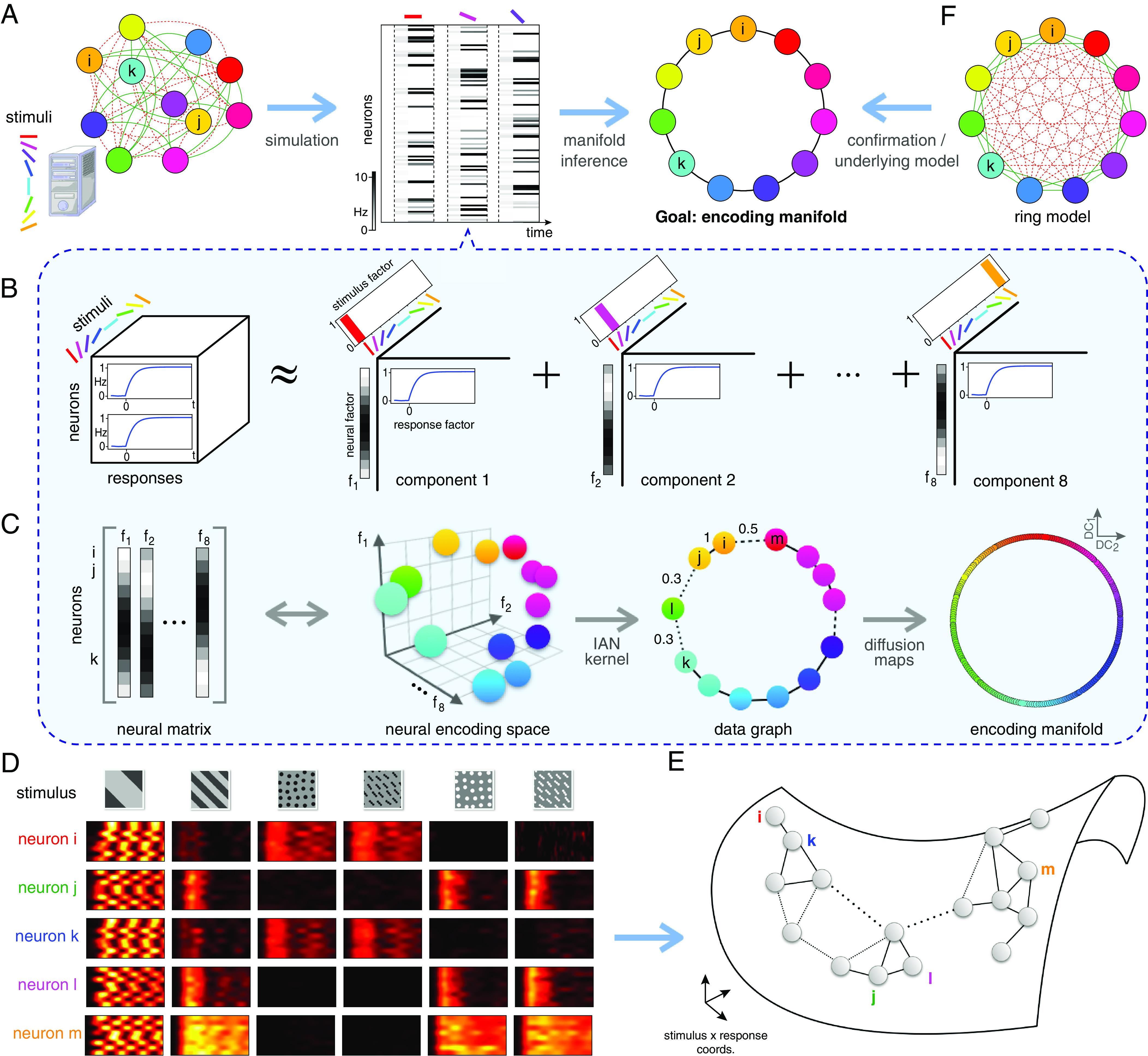
Illustration of the encoding manifold algorithm applied to an artificial example. (*A*) Suppose we are given an unknown circuit of orientation-selective neurons (*Left*). Stimulating it with orientated gratings yields responses; a spike train for each neuron for each stimulus trial (*Middle*). The goal is to use these data in an unsupervised manner to organize the neurons into a manifold: Each point on the manifold is a neuron and nearby neurons respond similarly in time to the stimulus ensemble (*Right*). (*B* and *C*) The manifold inference proceeds in two stages. (*B*) Stage 1: nonnegative tensor factorization (17) is used to multi-cluster groups of neurons by stimuli and by response. For this artificial example, all temporal responses are identical, so different neurons are organized by the different stimuli. Since eight orientations are used, eight latent components emerge (a procedure for selecting the number of components is described in *Materials and Methods*). (*C*) Stage 2: The neural factors are gathered into a matrix whose dimensions are #neurons×#factors; a row in this matrix represents the loading of each neuron in each factor $f_{i},i=1,2,\ldots ,8$. Equivalently, each row defines a vector in a stimulus/response-space, encoding the response of each neuron to each stimulus. A weighted data graph is then built from an iterated adaptive neighborhood similarity kernel (18) in this space and used with diffusion maps for manifold inference (19, 20). For this artificial example, the manifold is a ring in which neurons tuned to nearby orientations are neighbors. (*D* and *E*) Actual data are more complicated. To illustrate conceptually, five example RGCs and their responses to the stimuli are shown (*D*). Note that *i* and *k* respond similarly, so they should be close on the encoding manifold (*E*); neurons *i* and *j* respond differently, so they are distant on the manifold. Although *m* responds to the same stimuli as *j* and *l*, its response dynamics are different, so it is distant from the others. The underlying data graph is shown superimposed on the manifold. (*F*) The manifold produced from the artificial example (*A*) provides insight into the artificial problem we set up, namely the artificial ring model of orientation tuning from ref. 21.

To investigate and compare the organization of retinal and V1 population responses, we sought to organize the population responses on a manifold. Previous work has taken a “stimulus perspective” on these responses (10, 11, 13–16), producing a low-dimensional representation that organizes stimuli by the population response to each trial (Fig. 1*E*), allowing one to decode the identity of the stimulus that gave rise to the constellation of responses to a single stimulus trial. While these approaches are useful, our goal was distinct; we sought to identify how individual neurons contributed to signaling different stimulus features with respect to the entire neural population. Thus, we switched from the stimulus-based “decoding” perspective used previously (Fig. 1 *E* and *F*) to a neuron-based “encoding” perspective by developing an “encoding manifold” (Fig. 2). Each point on this encoding manifold is a neuron, not a stimulus, so it reveals how neurons are distributed in stimulus/response space. Thus neurons are organized by how they respond to features within the stimulus ensemble. These features included positive versus negative stimulus contrasts, high versus low spatial frequencies, motion in different directions, orientation, and any other features present in the stimulus set that drive different responses across the neural population. Neurons with similar feature selectivity and response dynamics will be nearby on the manifold, while neurons responding to different stimulus features or with distinct dynamics will be far apart on the manifold. Thus, the axes of this space are related to both stimulus features and temporal response characteristics. This approach differs substantially from the conventional one, which focuses solely on the stimulus selectivity of neurons. Here, we give equal emphasis on the dynamics of visual responses as to their stimulus selectivity. This dual focus is important for understanding how the brain processes visual input because the differences in dynamics are sufficiently large (tens of ms) that processing in higher visual areas will be strongly affected (22).

To illustrate the encoding manifold approach and validate that it 1) recovers the underlying functional structure of a neural population and 2) organizes these neurons appropriately on a manifold, we used the popular artificial ring model of orientation tuning (21, 23) (Fig. 2*F* and *Materials and Methods*). In this model, neurons tuned to nearby orientations excite one another while those tuned to different orientations inhibit each other. Equilibria of the network are the points on a ring (the space of possible orientations). We simulated responses of artificial neurons to low-frequency gratings drifting in eight different directions (Fig. 2*A*), each with the same dynamics. The encoding manifold was constructed in a two-stage procedure with no knowledge of the model’s underlying circuitry (Fig. 2 *B* and *C* and *Materials and Methods*): First, nonnegative tensor factorization (17) was used to relate stimuli to neural responses across the population of recorded cells. A neural encoding space was then constructed by bundling the neural factors into a matrix, in which a similarity kernel (18) could be defined. From this, diffusion maps—a non-linear inference algorithm (19, 20)—yielded the manifold. Expressed in diffusion coordinates, the ring emerged solely from the responses, even though the algorithm knew nothing of the underlying circuit organization.

In general, neural data will be more complex than that produced by a simple ring model (Fig. 2*D*), with many neurons exhibiting mixed selectivity (8, 24). As such, the manifold is likely to be richer than the ring, but the idea generalizes: Neurons with similar response profiles will be nearby on the manifold. A population of neurons with distinct responses will be far from other neurons on the manifold (Fig. 2*E*).

### Comparing Retina and V1.

**Fig. 3.**
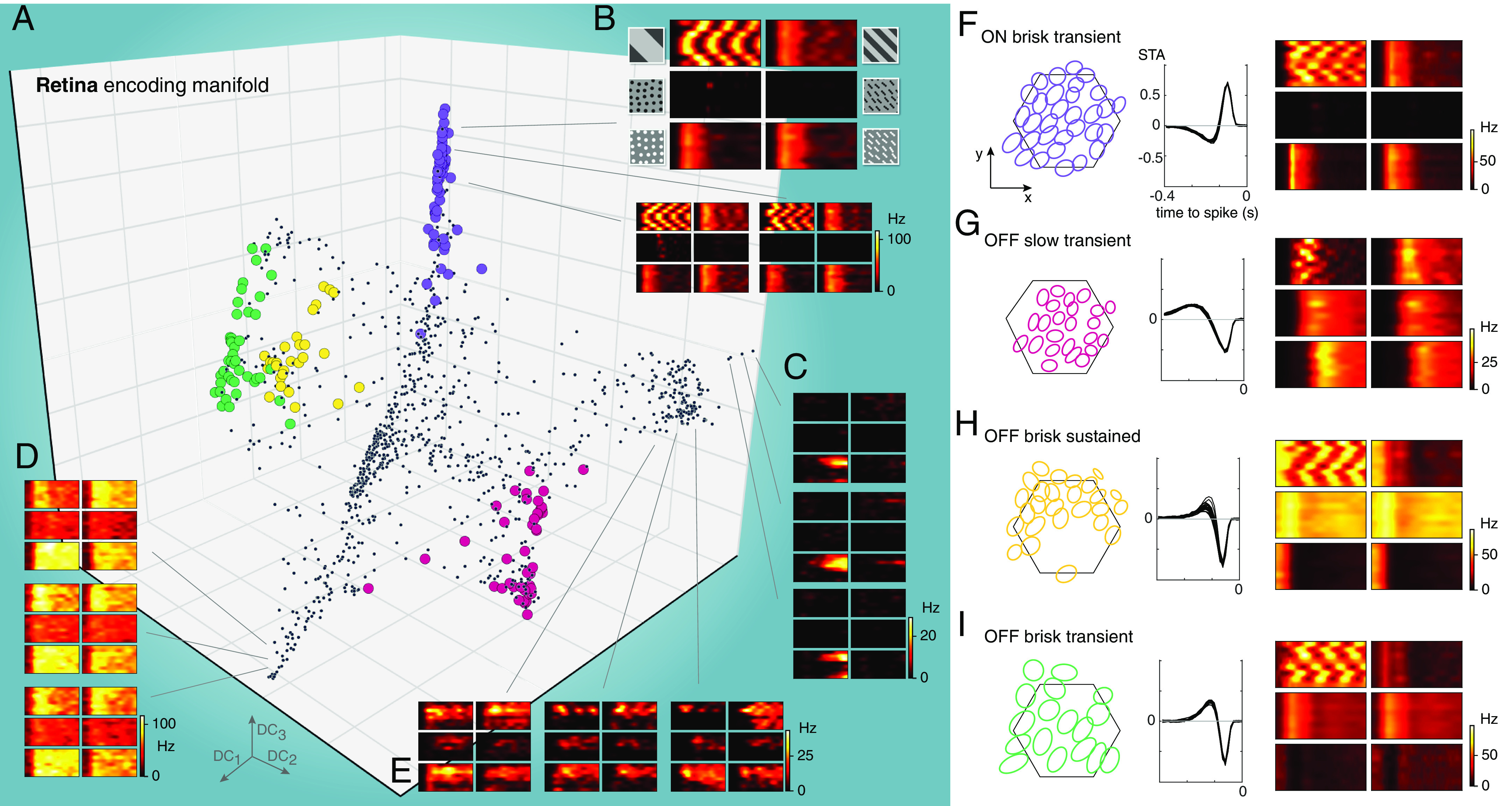
The encoding manifold formed by RGCs is clustered and organized by cell types. (*A*) Projection of the encoding manifold formed by RGCs onto the first three diffusion coordinates (*n* = 1,149 RGCs). (*B*) Three example RGCs from the purple cluster in *A*: Each sextet of PSTHs exhibits a delayed response to positive flows compared to negative flows and gratings (stimuli follow the same arrangement as in Fig. 1*D*). (*C*–*E*) Example individual RGC responses to the six stimuli from other locations on the manifold indicated by lines; they exhibit distinct response profiles, e.g., no response to negative flows (*C*) or direction-tuned (*E*). (*F*–*I*) The spatial receptive fields from each group of colored points form a mosaic-like arrangement that tiles space, confirming their identification as members of single classes of RGCs. *Left* panels show the 1-SD contour to a two-dimensional Gaussian fit to the spatial receptive fields estimated by computing the spike-triggered average to a checkerboard stimulus: Colors correspond to the points on the manifold in *A*, hexagon shows the outline of the electrode array. *Middle* panels show the temporal receptive fields from the spike-triggered average from the same cells in the *Left* panels. *Right* panels show an example sextet of PSTHs from a neuron in each mosaic to compare how the responses to the different stimuli vary across the different RGC types.

We first applied the encoding manifold approach to MEA recordings from 1149 mouse RGCs (Fig. 3 and Movie S1). Results from individual retinas were sufficiently similar that data from three retinas were combined to embed all data at once (*SI Appendix*, Fig. S3*D*). The resulting manifold exhibited clusters of neurons, with the cells in each cluster exhibiting similar response dynamics to similar stimuli. Other clusters, with different stimulus/response profiles, were relatively separated, so that following a trajectory along the manifold would reveal a population of nearly similar cells followed by an abrupt transition to another, different group of cells. See *Discussion* for further analysis of this manifold.

RGCs with nearly identical response properties would be expected to be nearby on the manifold. Since MEAs record large numbers of RGCs of some cell types more effectively and in larger numbers than other types (25, 26), we do not expect the number of clusters on the manifold to perfectly reflect the number of RGC types. Nevertheless, clear clusters from these data on the manifold did in fact correspond to distinct RGC types. To verify this, we examined the spatial receptive field locations of RGCs from individual retinas within each cluster by calculating the spike-triggered average to a checkerboard noise stimulus (27); this revealed that RGCs in a given cluster exhibited a mosaic-like organization (Fig. 3*F*–*I* and *SI Appendix*, Fig. S4). Mosaic organization is the hallmark of individual cell types in the retina (28–30), confirming that the manifold embedding could uncover many distinct RGC types. This observation serves further to validate, beyond the artificial ring model, the utility of the encoding manifold to reveal structure in a neural population.

**Fig. 4.**
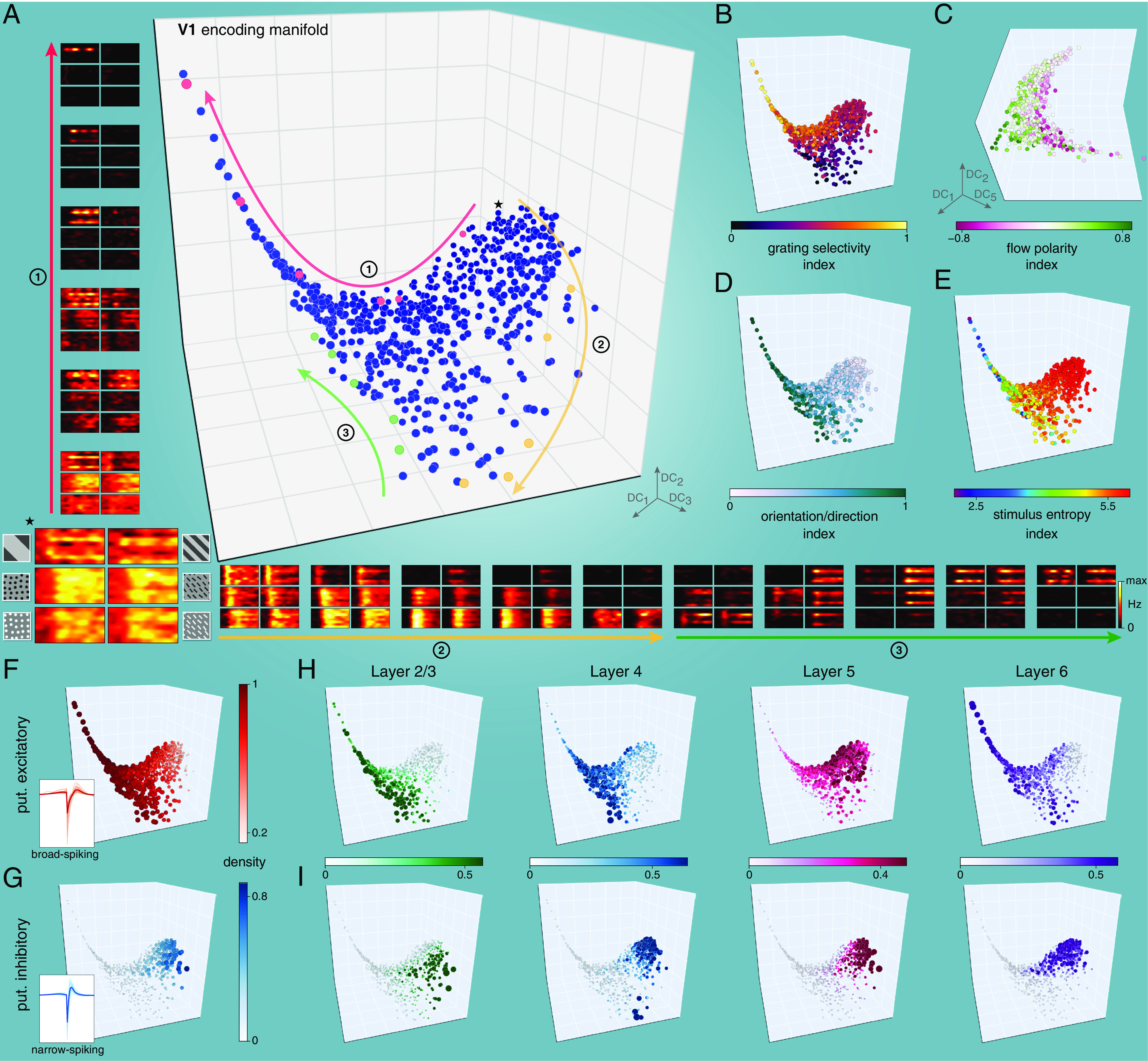
The encoding manifold formed by V1 neurons arranges neurons by their stimulus selectivity and response dynamics and is continuous rather than clustered. (*A*) Projection of the encoding manifold formed by V1 neurons onto the first three diffusion coordinates (n=640 neurons). The manifold consists of an extended arm formed by neurons tuned to low spatial frequencies connected to a higher-dimensional core of richly interconnected neurons. Instead of identifying clusters (as in Fig. 3), we follow paths over the manifold. Starting from the neuron indicated with ⋆, along path 1 (red arrow) cells transition from responding to all stimuli, to being highly selective for gratings moving in a particular direction (response profiles shown correspond to neurons highlighted in red). Along path 2 (yellow arrow, yellow neurons), neurons transition from responding to all stimuli to preference for positive-contrast flows. Along path 3 (green arrow, green neurons), neurons that are highly orientation selective to flows transition to neurons that respond to gratings of both low and high spatial frequency. Throughout, the transition is gradual, indicating a continuous manifold. (*B*–*E*) Visual feature selectivity (*Materials and Methods*) is well organized over the manifold; different regions correlate with particular features, but there are no apparent gaps. (*B*) Preference for gratings is highest along the arm. (*C*) A higher diffusion coordinate reveals that contrast polarity preference varies smoothly. (*D*) Orientation/direction selectivity declines toward the upper right. (*E*) Distributed stimulus selectivity increases toward the upper right. (*F* and *G*) Classification of neurons as putative excitatory or inhibitory (based on spike waveforms, see *Materials and Methods*) shows that physiological properties are also well-organized on the manifold. Different regions exhibit laminar specificity for both putative excitatory (*H*) and putative inhibitory (*I*) neurons, correlating with particular visual features from *B*–*E*. Neurons in *F*–*I* have color and size proportional to the local density of like-types in the data graph (*Materials and Methods*).

We next applied the encoding manifold approach to MEA recordings from 640 mouse V1 neurons (Fig. 4 and *SI Appendix*, Fig. S5*D* and Movie S2). The encoding manifold of V1 was quite different from that derived from the retina. Instead of clusters separated by abrupt changes, the V1 neurons were distributed relatively uniformly and continuously across the manifold (*Discussion*). To emphasize this difference, for the V1 encoding manifold, we speak of “neighborhoods” of cells, rather than distinct clusters, since preferred stimulus/response characteristics now vary smoothly from neighborhood to neighborhood.

While there are global coordinates (particular diffusion dimensions) that organize, e.g., contrast (Fig. 4*C*), a deeper picture emerges by following paths over the manifold. For example, Path 1 starts in a neighborhood of cells that respond to all stimuli before traversing an “arm” of cells selective only for low spatial frequency gratings (Fig. 4*B*). The origin of this path is a neighborhood populated primarily by fast-spiking putative inhibitory neurons (Fig. 4*G*), consistent with the observation that inhibitory neurons are broadly tuned (31). Excitatory neurons in layers 2/3 vs. layer 5 predominate in different regions on the manifold (Fig. 4*H*), consistent with the observations of mutual antagonism between these layers (32). For other aspects of how the manifold organization relates to neuroanatomy and physiology, see *SI Appendix*, Figs. S6 and S7.

The low spatial frequency arm is special; it consists mainly of excitatory neurons in layers 4 and 6, both of which, along with other layers, receive input from the lateral geniculate nucleus of the thalamus in the mouse (33). Because these cells are known to be tuned to orientation or direction of motion, we can ignore this feature, making the manifold insensitive to their actual direction preferences. This approach allows us to focus on other, less explored, properties. For that reason, we factored out direction preference with a permuted tensor factorization (*Materials and Methods*). This can readily be undone: When we isolated V1 neurons that responded primarily to low frequency gratings and that were orientation tuned, our embedding approach recovered a ring-like organization (*SI Appendix*, Fig. S8), reminiscent of the ring model of orientation tuning (Fig. 2*F*). This illustrates that we are sampling a wide assortment of orientation-tuned neurons in V1 and that they organize with similar orientations supporting each other. However, we emphasize that these neurons accounted for only 35% of the population, and only 17% of the population had low frequency gratings as the sole stimulus eliciting a statistically significant response (*Materials and Methods*).

The results above indicate that retinal output samples stimulus features in a relatively discrete manner, while V1 is substantially more continuous (*SI Appendix*, Fig. S10 *A* and *B*). This implies that cell diversity in the two circuits have conceptually different roles. In the retina, each RGC type produces a distinct selectivity for a constellation of visual features. In the cortex, cells appear to produce a continuum of stimulus selectivity that is relatively uniform across stimulus and response space, as one might expect either from a highly interconnected network or from a complete mixing of the parallel inputs from the retina. We discuss implications of, and caveats to, this interpretation below.

### Encoding Manifold for Convolutional Neural Networks.

**Fig. 5.**
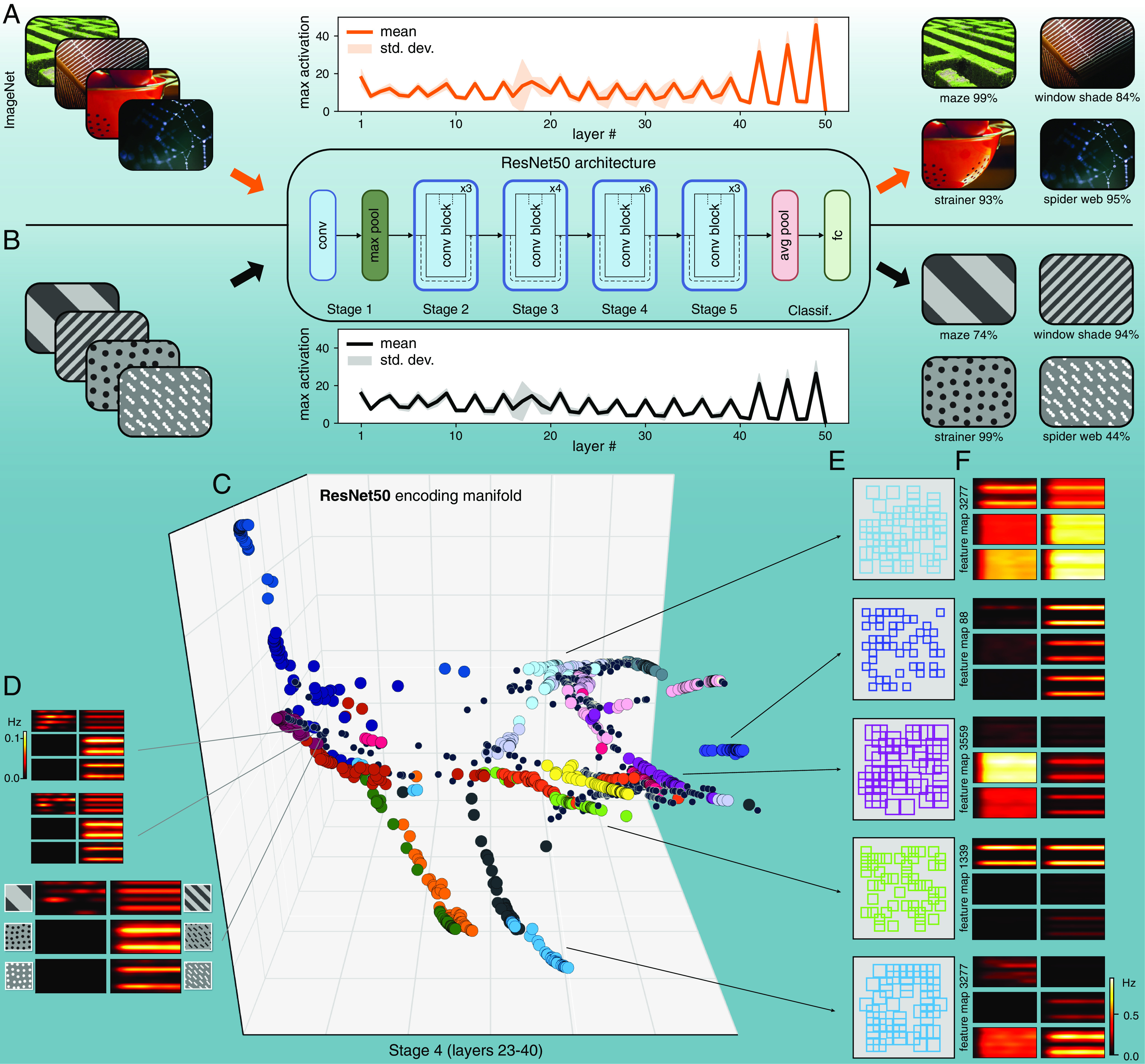
Encoding manifold of deep convolutional networks. (*A*) ResNet50 is a popular CNN pre-trained on ImageNet for image classification. (*B*) When applied to ResNet50, our stimuli (Fig. 1) generate similar levels of activation as those from natural scenes, and are also classified with confidence. (*C*) Encoding manifold computed from neuronal units from Stage 4, sampled at random in proportion to their activation (*n* = 2,000, *Materials and Methods*; other stages are shown in *SI Appendix*, Fig. S9). Color labels identify feature maps, showing that most neurons that share weights are grouped into well-defined clusters. (*D*) Although most individual neurons exhibit response profiles similar to those from V1, the topology of the manifold obtained is strikingly different (compare with Fig. 4). (*E*) Five examples of the spatial receptive fields (small squares) and (*F*) response profile centroids of groups of neurons belonging to the same feature map. These form clusters that tile visual space (cf. RGC types, Fig. 3) and have very specific feature selectivity, as expressed by their distinct response profiles.

Recently, convolutional neural networks (CNNs) have been widely used to model visual processing in the retina and cortex. While they recapitulate at least some features of visual processing, such as a hierarchical architecture that explicitly represents progressively more complex features in visual scenes, they also lack the dense recurrent connectivity present in most neural circuits (34–40). If CNNs were to accurately represent the way that populations of neurons encode visual features (41), the structure of their encoding manifolds should be similar to those found in biology. To test this, we constructed encoding manifolds from units in two popular deep CNNs: ResNet50 (42) (Fig. 5 and *SI Appendix*, Fig. S9 and Movie 3) and VGG16 (43) (*SI Appendix*, Fig. S10*D*). Since these networks were trained on ImageNet (44), we first established that our stimuli were classifiable and that they exhibited comparable activation levels across layers (Fig. 5*B*). Unlike V1, the encoding manifolds produced from CNNs were remarkably discrete (clustered) in their organization, even more so than the retina. For CNNs, most clusters corresponded to a feature map (set of units spanning position and sharing identical weights), reminiscent of RGC mosaics (Fig. 5*E*), revealing that activity patterns across feature maps were largely uncorrelated. These results indicate that CNNs do not encode visual feature space as neurons in V1 (and presumably other cortical areas) do, suggesting a crucial limitation to the use of CNNs for understanding cortical function (*Discussion*).

## Discussion

We began with a seemingly simple question: Whether populations of RGCs and cortical neurons sample visual space similarly. We developed a data-driven approach for topologically organizing how neurons in a circuit represent or encode stimuli by producing an “encoding manifold.” In this manifold, each point is a neuron and those with similar selectivity and response dynamics are nearby on it (Fig. 2). Remarkable topological differences emerged between the retina and V1: The retinal manifold was clustered (Fig. 3) while the cortical manifold was much more continuous (Fig. 4). When comparing these manifolds to that of CNNs, popular models of cortical visual processing, we found that CNNs exhibited a topology quite distinct from that of V1 and more clustered than that of the retina (Fig. 5). A density-based hierarchical clustering algorithm (*Materials and Methods*) confirms this qualitative assessment (*SI Appendix*, Fig. S10 *A*–*C*).

### Differing Functional Consequences of Cell Type Diversity in the Retina and V1.

Experiments beginning with Kuffler, Hubel, and Wiesel have demonstrated important differences in the responses of the retina and V1 (45, 46). However, those investigations have largely focused on differences in the optimal features that drive the cells (e.g., orientation tuning). Our purpose was different: to identify how stimulus space is sampled across each neural population and to develop a method to visualize and quantify these differences. As such, we have not focused on the stimuli that generate peak responses, but instead we have used the response dynamics of each neuron to a battery of stimuli to learn how the population collectively samples stimulus space. For example, it is possible that the retina and V1, while being sensitive to different visual features, could organize the encoding of these features similarly and thereby exhibit similarly clustered (or continuous) encoding manifolds. Furthermore, in the retina and V1, a revolution has recently occurred in our understanding of cell type diversity, driven by connectomics and transcriptomics (2, 47–51). In both retina and V1, there appear to be a large number of genuinely distinct cell types, rather than a continuum of morphologic and/or transcriptomic profiles. In the retina, it is clear these transcriptomic and morphologic cell types correspond (at least) nearly one-to-one with functionally distinct types (2). Our analysis recapitulates a “clustered” view in the retina, but produces a very different view of V1. Despite cell type diversity in both structures, the encoding manifold reveals a nearly continuous sampling of visual features in V1, while it produces a more clustered or discrete sampling in the retina. In the retina, the elongated clusters reveal only modest quantitative changes in the relative magnitudes of responses to the different stimuli, without changing the selectivity (*SI Appendix*, Fig. S4). In V1, on the other hand, there are several dimensions through which stimulus selectivity and response dynamics vary qualitatively and smoothly (*SI Appendix*, Fig. S6 and *Materials and Methods*, section 5); in contrast to the classical categorical distinction often imposed between simple and complex cells (52–54).

A potential caveat to the above conclusions is the choice of visual stimuli. We used stimuli spanning spatial frequency, contrast polarity, direction of motion, and orientation. Except for temporal frequency, this accounts for possibly the most commonly explored features in the vision literature. There are potentially a huge range of visual stimuli ($N_{s}$) to which one might measure responses and, of course, there are a large number of neurons that might respond variously to the different stimuli ($N_{n}$). In principle, there might be $N_{s}$ × $N_{n}$ distinct response types and because responses are extended in time this number is much larger. The encoding manifold is a simplified representation of this huge range of potential variation. To the extent that trajectories across the manifold correspond to distinct properties of visual stimulation, properties that we can make sense of, the manifold embodies an understanding of the real range of variation encoded in the neural circuit. This, combined with the fact that the stimuli we used also produced strong responses from artificial networks trained on natural scenes (Fig. 5*B*), leads us to conjecture that the topological differences we found should remain, even with richer stimulus ensembles. Nevertheless, expanding the stimulus set is an important direction for future research. One challenge is to produce stimuli that are at least approximately isotropic in space like flow stimuli (to mitigate the impact of cells having receptive fields at different retinotopic locations), yet retain naturalist structure (8). Features that could be added to our stimulus ensemble in a relatively straightforward way are 1) flow stimuli that temporally vary in their contrast or mean luminance, 2) superimposing flow stimuli moving at different speeds to mimic depth and parallax when animals move through natural scenes, and 3) including chromatic content. These stimulus variations would allow exploring how populations of neurons are organized with respect to contrast and luminance adaptation, depth, and chromatic tuning, all of which are exciting avenues for future work.

### Relationship between Manifolds and Neural Circuits.

**Fig. 6.**
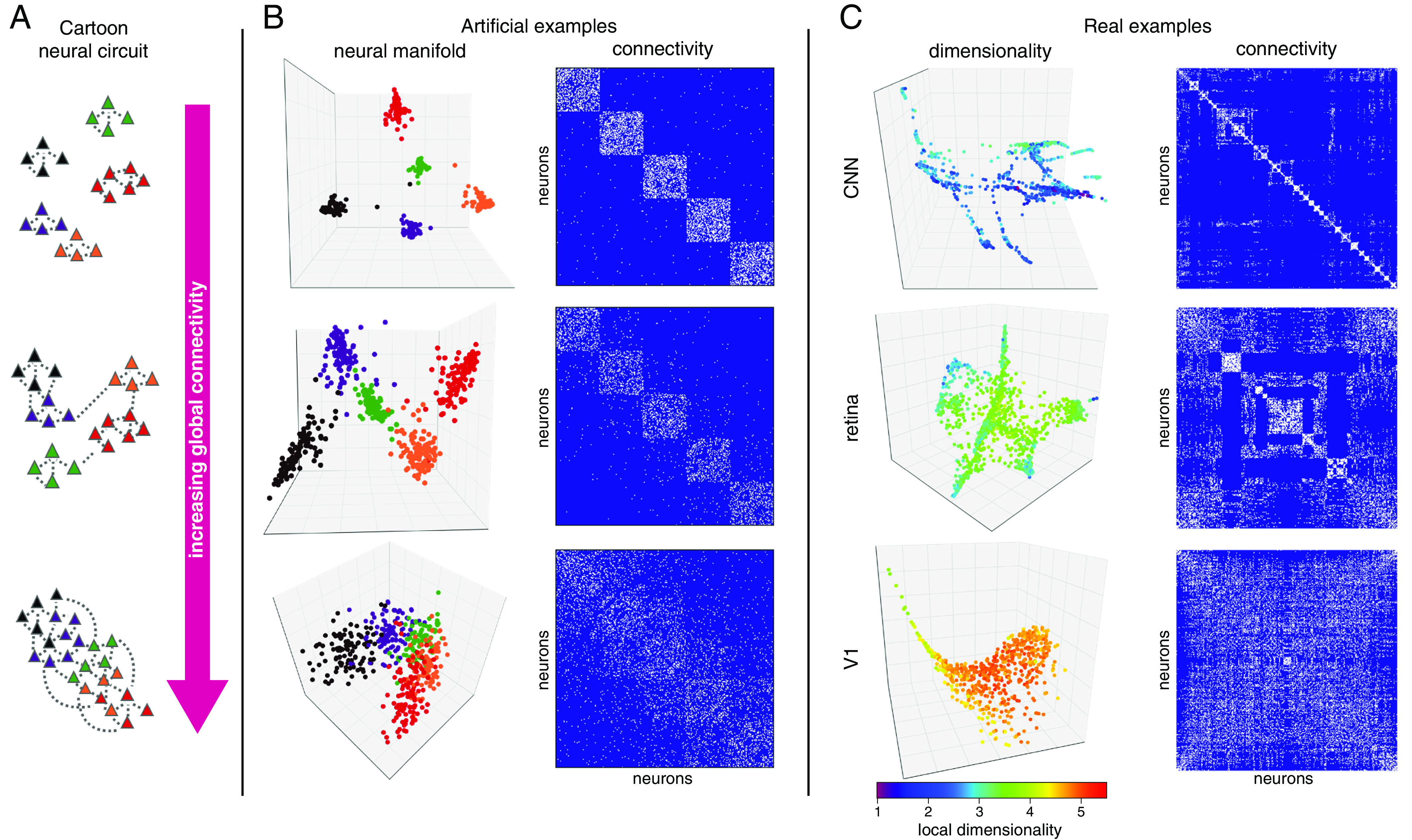
Circuits, connectivity, and dimensionality. (*A*) Three examples of cartoon neural circuits with increasing connectivity. (*B*) Extending the cartoon examples to artificial (stochastic block) models illustrates the relationship between the connectivity of the data graph and the resulting manifold. Neuron colors identify different blocks (*Left*); connectivity is represented by adjacency matrices with connections in white (*Right*). When dominated by intrablock connections, the manifold is disconnected (*Top*). As connections between blocks are added, the manifold becomes more continuous (*Bottom*). (*C*) Adjacency matrices for the data graphs producing the manifolds from Figs. 3–5 range from diagonally dominant (*Top*) to a more widespread connectivity (*Bottom*). V1 is continuous; the retina has clusters with some overlap; and deep convolutional networks are even more clustered. The manifolds are now colored by their local intrinsic dimensionality, which also increases from *Top* to *Bottom*, underlining how network complexity is higher in the cortex compared to the retina and CNNs (*Materials and Methods*, section 5).

We have established the relationship between encoding manifolds and the response properties of neurons to multiple stimuli via the construction of the data graph from our similarity kernel (Fig. 2*C*). Connections in this graph therefore represent similarity between neurons in response to the multiple visual stimuli. To illustrate what kinds of connectivity may lead to such different topologies, we consider the stochastic block model (55, 56) from social networks, a random model in which edges are more likely to be drawn between nodes belonging to the same “community”—or circuit (Fig. 6). If circuits are completely disjoint, the adjacency matrix (dimensions of which are neurons × neurons) has a block-diagonal structure: Units within a block are connected; those in different blocks are not (*Materials and Methods*). The resulting manifold embedding then consists of completely disconnected components, or clusters, as in most of our CNN examples (Fig. 5 and *SI Appendix*, Figs. S9 and S10*D*). As connections begin to couple these circuits, the manifold embedding begins to join components, until the connections become sufficiently numerous to mimic the more continuous V1 manifold. We should note that the stochastic block model perspective is only a start for guiding intuition; it is limited because it explicitly assumes the presence of blocks with uniform connection probability (57).

While our similarity kernel provides some information about potential connectivity, we emphasize it should not necessarily be one-to-one with the anatomical connectivity. The manifold represents neural activity, i.e., the responses to the stimulus ensemble; how such responses relate to the anatomical connectivity remains complex. For example, when neurons are nearby on the encoding manifold, it may be because those neurons receive common input (e.g., RGCs of the same type) or because they are synaptically connected to neurons with similar tuning and dynamics (e.g., the ring model), or both. Nevertheless, population-level inferences do arise from the encoding manifolds: Fig. 4 *H* and *I* and *SI Appendix*, Fig. S7 show a concrete example of how position on the manifold may relate to a specific anatomical location, such as the cortical layer.

### Comparison with Artificial Networks.

Recently there has been great interest in assessing how artificial deep networks compare to biological networks (58). This interest is motivated by the ability of CNNs to mimic many features of, for example, hierarchical visual processing found in macaques (34–40, 59). However, the process of building an encoding manifold revealed a very important difference between CNNs and V1. While individual units in a CNN may exhibit tuning like typical cortical cells, their encoding topology is completely distinct from a population perspective: instead of being nearly continuous like V1, they are highly clustered, where each cluster typically identifies with a feature map (akin to RGC mosaics). Surprisingly, even feature maps across successive layers are largely uncorrelated in their activity patterns, which explains the lack of continuity in the CNN encoding manifolds (*SI Appendix*, Figs. S9 and S10*D*). We have also applied this approach to two state-of-the-art CNNs used to model the mouse’s visual system: MouseNet (60), a network that is modeled directly on mouse neuroanatomy, and AlexNet(RI) (61), trained using contrastive learning. Although both studies reported high similarity with the population responses of neurons in the mouse cortex, the neural encoding manifolds produced for these networks (*SI Appendix*, Fig. S12) show clear concentrations of neurons (typically from the same feature map) interspersed by sparse regions. Therefore, their topologies are closer to those of other CNNs (*SI Appendix*, Figs. S9 and S10) and the retina (Fig. 3) than they are to V1. In future studies, the encoding manifold could be used for directly testing how further modifications to artificial networks, such as recurrence, may affect their encoding topology and similarity to V1 and/or other visual areas.

## Conclusion

In 1962, Hubel and Wiesel (46) noted that two novel response properties, not present in its inputs from the retina and dLGN, emerged in the visual cortex of the cat: orientation selectivity and binocularity. These properties were thought to be the product of the cortical circuit in combining the inputs it received. The absolute novelty of visual cortical responses is less clear in the mouse, with some degree of orientation selectivity and possibly binocularity in its inputs (62). However, the encoding manifold of mouse V1 reveals that, rather than preserving the qualitative distinctions evident in RGC responses, the cortex combines its parallel inputs to create a novel mode of organization: carpeting stimulus space, rather than discrete sampling.

The best way to compare visual areas within species, across species, or with artificial networks has been an important question. Our encoding manifold introduces a way to do this by providing a global as opposed to pairwise (63) view of how populations of neurons are organized to encode diverse stimuli. As we showed, it is applicable across areas and species.

Previous work claims similarity between CNNs and visual cortex, while our approach reveals qualitative differences. The distinct conclusions arise from differences in how the comparisons are made. Prior work compared response correlations among neurons to those among CNN units and found similar correlation structures (64). Our approach directly compares response dynamics across the population of neurons or the population of units in the CNN. When taking this perspective, real neural populations in the visual cortex are smoothly distributed in stimulus–response space, while units from CNNs are much more clustered.

Furthermore, labeling the neurons on the manifold with known anatomical properties permits inferences about how physiology correlates with visual feature selectivity; see *SI Appendix*, Fig. S7. Next questions implied by our results concern the dLGN and whether it is topologically more like the retina or the cortex, as well as extrastriate areas, for which there may be important differences in dimensionality and geometric properties. We believe this approach will also be useful for analyzing gene networks and the structure of other high-dimensional datasets.

## Materials and Methods

### Retina Experiments.

#### Animal procedures.

All retinal experiments were approved by the Duke University Animal Care and Use Committee. Adult mice (2 to 6 mo, C57Bl/6J, Jackson Laboratories, 000664) of both sexes were used. Animals were kept on a 12-h light/dark cycle with ad lib access to food and water. Prior to use, animals were dark-adapted overnight by placing the animal in a light-shielded box fitted with an air pump for circulation. Dissections were performed in complete darkness using infrared converters and cameras. Mice were decapitated, eyes enucleated, and placed into oxygenated room-temperature Ames solution (Sigma, A1420) during retinal dissection and vitrectomy as described previously (65).

#### MEA recordings.

A ∼1.5 × 1.5-mm piece from the dorsal retina was placed RGC side down on a MEA of 519 electrodes with 30-μm spacing (66–68). Oxygenated Ames solution perfused the retina throughout the experiment at a rate of 6 to 8 mL/min, heated to 32 °C. Hexagonal retinal recording array size in Fig. 3 and *SI Appendix*, Fig. S4 is 0.49 mm across and subtends about 15 degrees of visual angle.

#### Spike sorting.

Raw voltage traces from the MEA were spike sorted using YASS followed by manual curation (69, 70). Briefly, spikes were identified by events that crossed a threshold set to four SDs from the mean voltage. The electrical event 0.5 ms preceding and 1.5 ms following this threshold was extracted from the recording. These events were accumulated on each electrode. Projection pursuit was used to reduce the dimensionality of these signals and identify clear clusters of spikes. Putative cells with a spike rate >0.1 Hz and with <10% contamination estimated from refractory period violations were retained for further analysis.

#### Visual stimuli and receptive field measurements.

The image from a gamma-calibrated OLED display (Emagin, SVGA + XL Rev3) was focused onto photoreceptors using an inverted microscope (Nikon, Ti-E) and 4x objective (Nikon, CFI Super Fluor x4). Checkerboard stimuli were created and presented using custom Matlab code and presented for 30 min to estimate spatial and temporal receptive fields. The checkerboard stimuli were presented at a photopic light level (about 10,000 Rh*/rod/s), and a new checkerboard pattern was presented every 33 ms. Each square in the checkerboard stimulus was 75 × 75 μm. Custom software was used to present drifting gratings and flow stimuli at 60-Hz refresh rate, described below. RGC responses to checkerboard noise were used to estimate the spatial and temporal components of the spike-triggered average (STA) (27). The STA estimates the spatial and temporal integration of visual stimuli by the receptive field. Spatial receptive fields were fit with a two-dimensional Gaussian function to estimate the spatial extent of the receptive field center. A 1-SD contour of this fit was used to summarize the size and location of receptive fields of each RGC. The mean intensity of the grating and flow stimuli was also about 10,000 Rh*/rod/s.

#### RGC classification.

The time course of the temporal receptive fields, the autocorrelation function of the spiking dynamics and spatial receptive field size information were used to classify RGCs into different types, as described previously (68). Distinct functional types were confirmed by the formation of receptive field “mosaics” (30, 71): receptive fields that uniformly tile space and overlap at their 1-SD contour.

### Cortex Experiments.

#### Animal procedures.

Experiments were performed on adult C57BL/6J mice (age 2 to 6 mo) of either sex. All protocols and procedures are approved by the University of California–San Francisco Institutional Animal Care and Use Committee. Animals were maintained on a 12 h light/12 h dark cycle. Recordings were performed during the dark, more active phase of the cycle.

#### Preparation of mice for extracellular recording on the spherical treadmill.

Recordings were done on alert mice free to run on a polystyrene ball (200-mm diameter, Graham Sweet Studios) floating on an air stream from a single inlet at the bottom of a hemispherical bowl of slightly greater inside diameter. During recordings, the animal’s head is fixed to a rigid crossbar above the floating ball by screwing a titanium or stainless steel headplate cemented to the animal’s skull before recording using surgical procedures as described by Niell and Stryker (72). Following recovery from surgery for headplate attachment, the animal is allowed to habituate to the recording setup and learn to control the ball. Eye movements were captured under infrared illumination by a camera at the video frame rate of the stimuli (60 Hz). The centroid of the pupil was used to determine eye position.

#### Visual stimuli.

Visual stimuli were presented with gamma-corrected video display (Nanao Flexscan, 30 × 40 cm, 60 Hz refresh rate, 32 cd/m^2^ mean luminance, or Dell Ultrasharp 38 cd/m^2^ mean luminance) placed 25 cm from the mouse, subtending 60° to 75° of visual space. For current source density (CSD) analysis, we present a contrast-reversing square checkerboard (0.04 cpd, square-wave reversing at 1 Hz). Other visual stimuli were the same as those described for the retina experiments, and are described below. All stimuli variations were repeated 20 to 25 times according to a randomized sequence.

#### Extracellular recording in awake mice.

To carry out microelectrode recordings, a craniotomy was performed under brief isoflurane anesthesia, and the skull was thinned over a 1 to 2 mm diameter centered above the monocular zone of V1 (2.5 to 3 mm lateral to midline, 1 to 2 mm anterior to lambda). At least 1 h after full recovery from anesthesia, this small opening allowed insertion of a 1.1-mm-long double-shank 128-channel probe (73), fabricated by the Masmanidis laboratory through the NSF NeuroNEX program (University of California–Los Angeles). The electrode was placed at an angle of 30° to 45° to the cortical surface and inserted to a depth of 500 to 1,000 μm below the cortical surface. An additional period of 30 min to 1 h was allowed to pass before recording began. For each animal, the electrode was inserted no more than twice. Microelectrode and stimulus synchronization data were acquired using an Intan Technologies RHD2000Series.

#### Single-neuron analysis.

Single units in earlier experiments were identified using MountainSort (74), or in later experiments Kilosort 3 (75), in both cases followed by manual curation. For a few experiments in which the raw data that had been sorted with Mountainsort were later sorted with Kilosort, Kilosort found more than 90% of the same units plus 10 to 60% of additional well-isolated units. Data from 323 units from five experiments in three mice of the (8) report were combined with data from 317 units from 12 experiments in 12 mice to create the dataset used in the present report. Individual neurons were classified into broad spiking (putative excitatory) or narrow spiking (putative inhibitory) based on their extracellular spike waveform (ref. 76).

#### Cortical layer.

The cortical layer containing each isolated unit was determined using current source density (CSD) analysis on data collected during presentations of contrast-reversing square checkerboard. Briefly, extracellular voltages sampled at 20 kHz are bandpass filtered between 1 and 300 Hz to obtain local field potentials (LFPs) and then averaged across all 1 s positive-phase presentations of the checkerboard. Second spatial derivative of the average LFP traces along the length of the silicon probe provides us with the profile of CSD. The borders between layers 2/3 to 4, 4 to 5, and 5 to 6 are identified by spatiotemporal patterns of sinks and sources in the CSD plot (for example see figure 1C of ref. 77).

### Stimulus Ensemble.

To characterize neural responses with single-unit recordings, we presented interleaved drifting square-wave grating stimuli and flow stimuli moving in eight directions at a temporal frequency of 4 cycle/s and 50% contrast, with a trial duration of 1.25 s. Spatial frequencies used for gratings included 0.04, 0.15, 0.24, and 0.5 cycle/deg. As in ref. 8, we used flow stimuli with two different geometries. The first were nonoriented single-dot flows, and the other were oriented flow elements with three collinear dots. Both oriented and nonoriented variations had one version with positive contrast (white dots against a gray background), and another with negative contrast (black dots against a gray background). Dominant spatial frequency contents of 0.15, 0.24, and 0.5 cycle/deg were used, corresponding to the following dot diameters, in degrees of visual angle (respectively, approximate dot spacings, in multiples of diameter): 2.1 (2), 2 (1), 1 (1) for single dots; for 3-dot flows, diameter was divided by $\sqrt{3}$ to preserve the total area of each flow element. Because the flow stimuli were stochastic (in position and velocity), we presented at least three different instances of each variation, which were repeated to account for the desired number of total trials (at least 10 for the retina, and 20 for V1).

## Supplementary Material

Appendix 01 (PDF)

Movie S1. Animation of the retinal encoding manifold (Fig. 3) viewed from various angles, and zooming into one of the retinal ganglion cell clusters.

Movie S2. Animation of the V1 encoding manifold (Fig. 4) viewed from various angles, and showing neurons labeled by their grating selectivity index (see SI Methods).

Movie S3. Animation of the ResNet50 Stage 4 encoding manifold (Fig. 5), viewed from various angles, and zooming into a cluster of neuronal units from the same feature map.

## Data Availability

See supporting information, *Materials and Methods* for additional methods. Code for methods described in this paper and data used in this study are available at https://github.com/dyballa/NeuralEncodingManifolds (78).
