# Supplementary material for: Population encoding of stimulus features along the visual hierarchy
Source: Proc Natl Acad Sci U S A. 2024 Jan 16;121(4):e2317773121. doi: 10.1073/pnas.2317773121 (PMC10823231; doi:10.1073/pnas.2317773121)
Supplement: Supplementary file 1 — Appendix 01 (PDF) [file pnas.2317773121.sapp.pdf]

PNAS

Supporting Information for

Population encoding of stimulus features along the visual hierarchy

Luciano Dyballa, Andra M. Rudzite, Mahmood S. Hoseini, Mishek Thapa, Michael P. Stryker, Greg D. Field, Steven W. Zucker

Steven W. Zucker
E-mail: steven.zucker@yale.edu

This PDF file includes:

Supporting text
Figs. S1 to S12
Legends for Movies S1 to S3
SI References

Other supporting materials for this manuscript include the following:

Movies S1 to S3

Supporting Information Text

Methods

1. Embeddings of trials for decoding manifolds

To produce the decoding embeddings (Fig. 1f), we ran principal component analysis (PCA) using the implementation of the `scikit-learn` Python package (1) on a matrix whose dimensions were neurons by total number of trials. Each entry contained the average firing rate for that trial.

2. Ring model

The ring model of (2) is a model of an orientation hypercolumn, inspired by the primary visual cortex of cats and monkeys (3). It is used here as an example to illustrate the development of our approach (see Fig. 2). Neurons are parameterized by their preferred orientation (PO), θ, an angle between $-\pi/2$ and $\pi/2$. Both excitatory and inhibitory neurons ($n = 250$) form recurrent connections to all others, with the strength of interaction depending on the difference between their POs. The net interaction between neurons θ and θ' is given by $J(\theta - \theta') = -J_0 + J_2 \cos(2(\theta - \theta'))$, where $J_0 = 80$ is the amount of uniform inhibition, and $J_2 = 112$ the orientation-specific part of the interaction (see connection weight matrix in Fig. S1). The activity profile of the network at time t is given by

$$\tau_0 \frac{d}{dt} m(\theta, t) = -m(\theta, t) + g[h(\theta, t)], \tag{1}$$

where $\tau_0 = 5$ ms is a time constant, and g a semilinear gain function with threshold $T = 1$ and gain factor $\beta = 0.1$ (refer to (2) for details). All neurons receive external input expressed as $h^{\text{ext}}(\theta) = c[1 - \epsilon + \epsilon \cos(2\theta)]$, where $\epsilon = 0.5$ regulates its angular anisotropy and the coefficient $c = 1.9$ sets the stimulus contrast. The total synaptic input at time t of a neuron θ is

$$h(\theta, t) = \int_{-\pi/2}^{\pi/2} \frac{d\theta'}{\pi} J(\theta - \theta') m(\theta', t) + h^{\text{ext}}(\theta - \theta_0), \tag{2}$$

where θ_0 is the orientation of the external stimulus.

After receiving an oriented input (the external stimulus), each artificial neuron responds proportionally to the similarity between the orientation of the stimulus and its own preference. Its ultimate change in output (treated as spikes/s) is obtained by integrating the inputs from all its neighbors plus the external input. In our simulation, 8 different stimuli were used with orientations evenly distributed between $-\pi/2$ and $\pi/2$, and a trial length of 1.25 s. Each stimulus was presented 10 times, with a Gaussian noise stimulus (std. dev. 3) used during the ISI to make the response in each trial essentially unique, given that the model is deterministic.

3. Data analysis: tensor decomposition

This section is organized as follows. We begin with several essential points about tensor factorization (Fig. 2b), then specify our tensor explicitly. For general background, see (4). Next, we show how the neural factor matrix can be used to define the neural encoding space (Fig. 2c), and address the question of how many tensor factors to use. These are essential steps that will allow us to define similarities between neurons and infer the neural encoding manifold (Fig. 2).

Tensor decomposition. In a tensor CP decomposition (5, 6), an n-way tensor $\boldsymbol{T} \in \mathbb{R}^{I_1 \times I_2 \times \ldots \times I_n}$ is approximated by a sum of rank-1 tensors. Let R be the number of components chosen; then, following the notation of (7), we may express this for the specific case of a 3-way tensor as:

$$\tilde{\boldsymbol{T}} = [\![X^{(1)}, X^{(2)}, X^{(3)}]\!] \equiv \sum_{r=1}^{R} v_r^{(1)} \circ v_r^{(2)} \circ v_r^{(3)}, \tag{3}$$

where $\circ$ stands for the vector outer product and $X^{(k)}$ is called a *factor matrix* containing the factors $v_r^{(k)}$ as its columns. Each rank-1 tensor is formed by the outer product between each factor in the same component (following the convention that a *component* refers to each set of associated factors, one from each tensor mode).

It is often convenient to normalize factors to unit length and collect their original magnitudes into a single scalar for each component, $\lambda_r = \prod_{k=1}^{n} \|v_r^{(k)}\|$, where r indexes the component and k the factor mode, for an n-way tensor. Combining all scalars into a vector $\lambda \in \mathbb{R}^R$, we may rewrite eq. 3 as

$$\tilde{\boldsymbol{T}} = [\![\lambda \,;\, X^{(1)}, X^{(2)}, X^{(3)}]\!] \equiv \sum_{r=1}^{R} \lambda_r v_r^{(1)} \circ v_r^{(2)} \circ v_r^{(3)}. \tag{4}$$

Most algorithms for CP decomposition use the squared reconstruction error as objective function (8):

$$\min_{X^{(1)}, X^{(2)}, X^{(3)}} \frac{1}{2} \|\boldsymbol{T} - \tilde{\boldsymbol{T}}\|^2. \tag{5}$$

where $\|\cdot\|$ is the norm of the vectorized tensor (analogous to the Frobenius norm for matrices). Since the data consist of neuronal firing rates, we compute a non-negative tensor decomposition, also known as non-negative tensor factorization (NTF) (9), formulated as in eq. 5 with the additional non-negativity constraint $X^{(k)} \geq 0, \forall\, k$. In contrast to the negative version, it achieves a parts-based representation with more easily-interpretable components (8, 10); moreover, it can produce more stable factors, is less likely to overfit, and has comparable parameter efficiency (4). To compute the decomposition, we use the gradient-based direct optimization approach (OPT) (11) via the Tensor Toolbox implementation (12), adapted to allow permutation of the response factors (see below).

Items to note: the factor matrices are not orthogonal and may in fact have linearly dependent columns. Importantly, the best rank-r factorization may not be part of the best rank-$(r-1)$ factorization (13). Because tensor factorization requires an initial guess for the factors, each run may yield different results, even when the same number of components is chosen. Reconstruction error is commonly normalized by the Frobenius norm of the original tensor (e.g., 4), and the values shown in Figs. S2d, S3c, S5c, S8e follow this convention.

Tensor of neuronal responses (Fig. 2b). We utilize a 3-way tensor having neurons, stimuli, and responses as modes. We choose not to use individual trials since there is no expectation of significant change in the responses over different repetitions of the stimulus (in contrast with, e.g., a learning task). Moreover, by using stimuli as a mode, we obtain more interpretable factors that directly relate to parts of the stimulus ensemble. For each neuron recorded, and stimulus used, we have a response which could be either a 1-D response curve—as was the case for the ring model (Fig. S1) and the embedding of V1 using low frequency gratings only (Fig. S8)—or a 2-D response map (stimulus direction × time; see Fig. 1). The latter was used in vectorized form (concatenation of the responses to the 8 directions of motion), thus forming a 3-way tensor.

The responses of a neuron to each stimulus were kernel-smoothed using the Improved Sheather-Jones algorithm for automatic bandwidth selection (14), which does not assume normally distributed data; we used the `KDEpy` Python implementation(15). For the bandwidth fitting, we combined all trials in which the neuron was responsive (at least 5 for retina, and 15 for V1). Kernel bandwidths were allowed to be automatically selected within the range 10–50 ms, using the stimulus direction with the highest number of spikes.

Neural encoding space (Fig. 2c). Next we define a matrix formed by the neural factors obtained from NTF, and define the space associated with it, from the perspective of linear algebra. We refer to this as the neural encoding space (see Fig. 2c). From eq. 4, we can express the reconstruction of an individual data point, x_i, as:

$$\tilde{x_i} = \sum_{r=1}^{R} \lambda_r v_r^{(1)} \circ v_r^{(2)} \circ \ldots \circ v_r^{(n)}, \tag{6}$$

and for the entire tensor as:

$$\tilde{\boldsymbol{T}} = X^{(1)} \Lambda \left(X^{(2)} \circ X^{(3)} \circ \ldots \circ X^{(n)} \right), \tag{7}$$

where Λ is the diagonal matrix with entries $\Lambda_{rr} = \lambda_r$.

Since the neural factors are represented by the first mode of the tensor, we henceforth denote $X^{(1)}$ by $\mathcal{N}$. Now note that the outer product between the vectors of the remaining modes of each component is a $(n-1)$-way tensor, which, if vectorized, becomes a unit-norm vector, and can be considered a basis vector. Technically, this collection of vectors (one for each component r) represents a *frame* (16), since in general it will be redundant (containing more vectors than the true dimensionality of the space that they span). We refer to it as the *stimulus-response frame*.

We can then define a matricized version of $\boldsymbol{T}$ with respect to the first mode, denoted by $X_{(\mathcal{N})} \in \mathbb{R}^{(I_2 \times \ldots \times I_n) \times I_1}$, as:

$$X_{(\mathcal{N})} \approx B \Lambda \mathcal{N}^{\top} \tag{8}$$

with each column $b_{:,r}$ of B given by

$$b_{:,r} = \text{vec}\left(v_r^{(2)} \circ v_r^{(3)} \circ \ldots \circ v_r^{(M)} \right) \tag{9}$$

for $r = 1, \ldots, R$. These R columns can therefore be regarded as unit-norm basis vectors. So each data point $\boldsymbol{x}_i$ (column of $X_{(\mathcal{N})}$) is approximated as a non-negative linear combination of the vectors in the stimulus-response frame.

In this case, it is convenient to multiply each column $v_r^{(1)}$ of $\mathcal{N}$ by its corresponding λ_r and work with a scaled neural matrix, $\mathcal{N}_\lambda$:

$$\mathcal{N}_\lambda \equiv \mathcal{N} \Lambda. \tag{10}$$

The matrix $\mathcal{N}_\lambda$ thus contains all information needed to reconstruct each neuron $\boldsymbol{x}_i$ in terms of the stimulus-response frame: its linear combination coefficients are given by the ith row of the neural matrix $\mathcal{N}_\lambda$. The dimensionality of the data points is thus reduced to R. We now discuss the procedure for finding R.

Choosing the number of tensor components. The neural matrix depends on the number of components, F, a choice that is challenging and computationally costly (17). The standard procedure would be to minimize reconstruction error, where one would use the number of components sufficient to meet a prespecified tolerance. Our goal is different, however: We seek to find those features that maximally organize the data. That is, we are concerned with the span of the latent space and the stability of the neural matrix. We now develop this idea.

When F is too small, there are not sufficient factors to capture all the relevant structure present in the data. When it is too large, degeneracy of the resulting components may arise, a phenomenon known as overfactoring (11): some factors could mostly capture noise, and otherwise meaningful factors could be split. Because in NTF the factors are in general not orthogonal, some degree of redundancy typically exists, i.e. the factor matrix is overcomplete. Applying PCA to the neural matrix reveals its redundancy by re-expressing it in terms of an orthonormal basis formed by the eigenvectors of the sample covariance matrix. Its eigenvalues express the variance of the data when projected onto each eigenvector. Looking at this spectrum allows one to estimate the rank of $\mathcal{N}$, or the intrinsic dimensionality of the latent neural space. Intuitively, while more factors should explain more variance, at some point the gain can become insignificant or a product of overfactoring. By analogy with overfitting, we argue that the best F is the smallest one that maximizes the variance explained by the neurons in this low-dimensional subspace. As we now show, this choice is achievable.

An example factorization is that of the ring model from Fig. 2: each component is responsible for a single stimulus, and neurons overlap slightly as one moves from one component to the next (Fig. S1). This is predictable from the simulation setup: each neuron's response function decreases slowly as a function of the difference between their preferred orientation and the orientation of the input stimulus. Now since all neurons respond with the same temporal dynamics (minus noise), only a single response pattern is required to reconstruct the responses to any of the 8 stimuli in the original tensor. Therefore F=8 is necessary and sufficient, regardless of the number of neurons used. We now generalize from this example.

Intuitively, one might want F to maximize the span of the neural factors. However, since the factors are in general not orthogonal, this number might greatly exceed the intrinsic dimensionality of the data. When each of the neural factors has unit norm, the spectrum of its covariance matrix reflects how much redundancy exists in the frame, so instead of using the span directly, we use the nuclear norm (sum of eigenvalues, or variances) as a measure of the (putative) rank of $\mathcal{N}$. This measure is widely used in matrix completion, since it is the convex relaxation of rank (18). To compare across different choices of F, then, we normalize the variance spectrum by its maximum eigenvalue (since uniformly scaling the data points will not change the embedding). This approach is illustrated in Fig. S2 for the ring model. In particular, notice how the nuclear norm tends to increase rapidly for small F, then plateaus over a short region (Fig. S2d, highlighted in gray) before increasing again. Similar behavior obtains for real data (note the gray regions in Figs. S3b, S5b, S7d, S8d, and S9b). We choose a point ($F = R$) from this region, representing the relaxed rank for $\mathcal{N}$. Experimentally we verify that varying R within this region yields similar results. As F increases further, the total variance tends to keep increasing albeit at a different rate, since the resulting components either overfit or break down pre-existing factors (11).

One detail remains with regard to NTF, since a random initialization is used in the algorithm. We use 50 different initializations for each value of F, and choose the best-fit model (minimum reconstruction error) within $F = R$. An alternative is to use the average weighted graph from all different initializations; it produced similar results.

Permuted tensor decomposition. As indicated in the main text, normalizing the neurons' preferred direction of motion allows the manifold inference to focus on other, less explored, properties. For example, two neurons narrowly-tuned to orientation can be considered to be similar regardless of their particular preferred orientation (in Fig. S8 we show how orientation or direction preferences can be considered explicitly). Thus for the retina, V1, and deep networks datasets we develop an algorithm in which the decomposition can automatically search through alternative versions of the data tensor to minimize reconstruction error. This allows one to solve for factors that are agnostic to the actual orientation/direction preference of each neuron. The direct optimization (OPT) method from (11) is well-suited for this because it solves the CP decomposition using gradient descent, solving for all factor matrices simultaneously (as opposed to traditional methods using alternating least squares (5, 6)). As shown below, this makes it feasible to incorporate permutations of entries along one of the tensor modes directly into the optimization loop. In the above datasets, because the stimuli drifted in 8 different directions, such a strategy amounts to computing each step of the decomposition as the argmin of all possible circular-rotations of the rows in each of the 2-D response maps in $\boldsymbol{T}$. As described above, since the response maps are vectorized to become tensor fibers, the circular-rotation effectively becomes a permutation of their entries.

Following (11), the objective function of eq. 5 can be expressed as three summands:

$$\begin{aligned}\min f(\boldsymbol{T}, X^{(1)}, X^{(2)}, X^{(3)}) \equiv \\ \frac{1}{2}\left\|\boldsymbol{T}\right\|^2 - \left\langle \boldsymbol{T}, [\![X^{(1)}, X^{(2)}, X^{(3)}]\!] \right\rangle \\ + \frac{1}{2}\left\|[\![X^{(1)}, X^{(2)}, X^{(3)}]\!]\right\|^2,\end{aligned} \tag{11}$$

where the second summand is the inner product between $\boldsymbol{T}$ and its CP approximation.

At any given step t of the optimization, let its current factor matrices be expressed as $[\![X^{(1)}, X^{(2)}, X^{(3)}]\!]^{(t)}$, and denote a permuted version of $\boldsymbol{T}$ as $\boldsymbol{T}'$. To enforce the desired invariance, we shall replace $\boldsymbol{T}$ with the optimally permuted tensor, $\boldsymbol{T}^{\star^{(t)}}$, namely that obtained from the set of independent permutations of each individual response map in $\boldsymbol{T}$ resulting in the lowest

value of f:

$$\boldsymbol{T}^{\star(t)} \equiv \operatorname{argmin}_{\boldsymbol{T}'} \quad f\left(\boldsymbol{T}', [\![X^{(1)}, X^{(2)}, X^{(3)}]\!]^{(t)}\right). \qquad [12]$$

To find $\boldsymbol{T}^{\star(t)}$, it suffices to compare the value obtained for the second summand in eq. 11 across all permutations (since the norm of $\boldsymbol{T}$ is unchanged by the permutations). Likewise, in computing f at step t, only that term is modified:

$$\begin{aligned} f^{(t)} =& \frac{1}{2}\|\boldsymbol{T}\|^2 - \left\langle \boldsymbol{T}^{\star(t)}, [\![X^{(1)}, X^{(2)}, X^{(3)}]\!]^{(t)} \right\rangle \\ &+ \frac{1}{2}\left\| [\![X^{(1)}, X^{(2)}, X^{(3)}]\!]^{(t)} \right\|^2. \end{aligned} \qquad [13]$$

Finally, the corresponding gradient of $f^{(t)}$ can be computed by replacing $\boldsymbol{T}$ with $\boldsymbol{T}^{\star(t)}$ in eq. 4.9 from (11). The overhead of finding the optimal permutation is typically reasonable, because the tensor norm in the objective is computed elementwise. Thus the optimal permutation of each response map can be found independently. This results in an upper bound on scaling the computational cost of the objective by a constant factor, namely the number of permutations allowed (in the case of our stimuli, 8).

Fitting statistically non-significant responses. Responses to each stimulus were tested for significance by performing a two-tailed Mann-Whitney test comparing the mean activity during the second half of the ISI immediately preceding the stimulus versus mean activity over any interval of the same length within the stimulus presentation. This strategy allowed transient responses to be able to reach the same level of significance as sustained responses. Responses were deemed significant if they had $p < 0.0005$. When choosing between medium and high spatial frequency stimuli for each neuron, the optimal frequency was chosen as that which produced the largest number of significant stimuli among gratings, positive flows (1- and 3-dot), and negative flows (1- and 3-dot). Low spatial frequency gratings, because they had no flow counterpart at an equivalent frequency, were always included. See Material and Methods, Visual Stimuli for more details.

Responses that were not statistically significant (see below) were removed (zeroed) from $\boldsymbol{T}$ before NTF to prevent introducing excessive noise. However, for the scheme to be able to reconstruct all data, after NTF we reintroduced these non-significant responses (see below). Responses that are truly non-significant are less reproducible and have very low magnitudes, so their neural coefficients will be close to zero. On the other hand, responses that are selective but too weak to pass the statistical test are likely to be well fitted regardless, since their response patterns will match those of other (significant) neurons. Moreover, using only highly significant responses in NTF ensured the factors obtained were interpretable and consistent.

Tensor normalization. We now discuss several details regarding the preprocessing of neural responses to construct the tensor. Since the objective of NTF is to minimize reconstruction error (eq. 5), response patterns with longer periods of activity (or with larger area over the response map) will have a higher reconstruction priority in terms of cost minimization compared to shorter responses. This creates a bias towards producing factors that reconstruct the sustained response patterns. We prevent this by pre-normalizing each stimulus response to have unit norm in $\boldsymbol{T}$.

Once the factorization is computed, the relative activity levels of a neuron to each stimulus can be reintroduced by rescaling the unit-norm responses by their original relative magnitudes. This is done by re-fitting the neural coefficients in $\mathcal{N}_\lambda$ to the rescaled responses using linear least squares. The rescaled tensor used in this step includes the non-significant responses (see above).

After this step, we reach a concrete interpretation for the neural coefficients: when a stimulus factor s_i includes a single stimulus, its corresponding neural factor n_i encodes the relative magnitude of the response pattern p_i for each neuron in the population. Finally, for this to hold also in cases in which the same s_i contains multiple stimuli, we make the following rebalancing between n_i and s_i. Let s_i^{max} by the maximum value in s_i. Then, replace n_i with $n_i' = s_i^{max} n_i$ and s_i with $s_i' = s_i / s_i^{max}$. Additionally, we take their square-root so that the magnitude of the coefficients for each stimulus (when they are separable) approximates its relative response magnitude. This matters since the neuron organization should reflect not only the response patterns but also their stimulus preference in terms of relative firing rate.

4. Data analysis: neural encoding manifolds

Low-dimensional embeddings (Fig. 2c, right). Manifold embeddings were obtained by first computing the IAN similarity kernel (19) from the pairwise distance matrix obtained from the PCA projection of the neural matrix as input (see above). We used the number of principal components necessary to explain at least 80% of the variance. IAN uses a multiscale Gaussian kernel where the individual scales (or neighborhood sizes) are automatically inferred based on the local geometry of the data; its default parameters were used throughout (19). The converged weighted graph (similarity matrix) from IAN was then used as input to the diffusion maps algorithm (20, 21), a nonlinear spectral embedding method based on the eigendecomposition of the normalized graph Laplacian. Diffusion maps were preferred over other popular methods (e.g., 22, 23) because it is a deterministic algorithm and tends to better preserve the original topology of the data, cf. examples in (19). The standard parameters $\alpha = 1$ (Laplace-Beltrami approximation) and diffusion time $t = 1$ were used. It produces a set of diffusion coordinates which can be used to embed the neurons in stimulus-response coordinates (see above). When data graphs were disconnected, a high number of coordinates were required to embed all components together. To aid visualization, in these cases, we projected the diffusion distances between points using classical multidimensional scaling.

Layer and type densities and encoding similarity. Laminar and putative type densities for each cortical cell (Fig. 4) were computed as the fraction of adjacent nodes in the non-weighted IAN graph belonging to the same putative type and layer. The neuron densities were then arranged into a vector for each layer. Encoding similarities were computed as the non-negative cosine similarity between non-negative z-scores of the density vectors for different types and layers (Fig. S7).

5. Data analysis: intrinsic dimensionality

Intrinsic dimensionality (ID) plays an important role in manifold learning as a means to describe the degrees of freedom within the volume occupied by the data in ambient space, and it has been applied to the decoding approach (24) (Fig. 1f). In an encoding manifold, ID can be interpreted as the number of different directions of progressive change in stimulus-response space. For example, in the ring model the manifold is one dimensional, because it has a single latent variable, namely orientation, even though it is originally within a much higher-dimensional ambient space (the number of stimuli times the number of time points). Therefore, starting from a given neuron, orientation is the only variable that characterizes change when moving along the manifold since, for this artificial data, dynamics are constant. In CNNs, these latent directions correspond largely to variations in response magnitudes due to position over the feature map. In the retina, directions signify variation in stimulus preference, but these are highly constrained (Fig. S4). Across most of the V1 manifold the response profiles vary smoothly in several different ways, corresponding to different directions over the manifold (Fig. S6). The sole exception to this is the region where neurons respond only to low spatial frequency gratings, where the ID is mostly due to phase and tuning width differences, and direction vs. orientation tuning. Importantly, responses can vary only in some directions, and not others. One example is the absence of neurons with high orientation selectivity to 3-dot flows with a particular contrast polarity preference.

Importantly, even though the actual ID may vary with the variety of the stimulus set used (25), we believe that the ID of these three networks reflects intrinsic properties of their circuits, and conjecture that they may be robust to variations in the stimulus set. To estimate the ID of the manifolds, we used the neighborhoods of the unweighted IAN data graph as input to the Neighborhood Correlation Dimension algorithm (19), which can estimate the dimensionality around each data point (shown in Fig. 6).

The adjacency matrices from Fig. 6 had the neurons sorted according to the dendrograms produced using the hierarchical clustering algorithm HDBSCAN (26, 27), shown in Fig. S10a–c. This algorithm is popular (28–30) because it realizes the intuitive notion that local concentrations of points, i.e., regions of higher density, indicate clustering. Its main parameter is the number of nearest neighbors, k; to avoid having to choose an arbitrary value for k, we instead use the neighborhoods inferred by the IAN kernel (section 4). Importantly, for the cortex manifold, HDBSCAN indicates an absence of clusters (Fig. S10a). This contrasts directly with other approaches that assume *a priori* a clustered organization to the data (31–33). Here, the clustering assignment is used for confirmation of the observed topology.

6. Data analysis: stimulus selectivity

Selectivity indices for various visual features (Fig. 4b–e) were computed as follows. Let the maximum response (instantaneous mean firing rate) to any of the grating stimuli (flow stimuli, respectively) be denoted as $G_{\max}$ ($F_{\max}$, resp.). A *grating selectivity index* for a given neuron was computed as the ratio $G_{\max}/(G_{\max} + F_{\max})$.

Let the relative response magnitude of a neuron to a given stimulus be defined as the ratio between its maximum response to that stimulus and its maximum response to any stimulus (therefore yielding a number between 0 and 1). Defining P_{rel} (N_{rel}, resp.) as the relative magnitude of a neuron's response to any positive contrast flow (negative contrast flow, resp.), a *flow polarity index* was computed as the difference $P_{\text{rel}} - N_{\text{rel}}$.

An orientation selectivity index (OSI) was defined as: $(R_{\text{pref}} - R_{\text{ortho}})/(R_{\text{pref}} + R_{\text{ortho}})$, where $R_{\text{pref}} = (R_{\text{peak\_dir}} + R_{\text{peak\_dir}+\pi})$ is the response (average firing rate) for the preferred orientation and $R_{\text{ortho}} = (R_{\text{ortho\_dir}} + R_{\text{ortho\_dir}+\pi})$ is the response for the orientation orthogonal to the preferred one. A direction selectivity index (DSI) was defined as: $(R_{\text{pref}} - R_{\text{null}})/(R_{\text{pref}} + R_{\text{null}})$, where R_{pref} is the response (average firing rate) for the preferred orientation and R_{null} is the response for the null orientation (π rad apart from the preferred one). An orientation/direction selectivity index (ODSI) was defined as the maximum value between a neuron's OSI and its DSI.

A stimulus entropy index was defined as 2^H, where H is the base-2 entropy of the vector containing the relative response magnitudes of a neuron to the 6 stimulus classes used in the experiments (divided by their sum). It therefore ranges between 0 (case in which the neuron responds to a single stimulus) and 6 (when it responds with uniform magnitude to all stimuli).

Cortical cells were classified as well-tuned to low-frequency gratings by fitting their direction tuning curves to a double Gaussian, following the fitting method of (34) and selecting those cells with small fitting error, followed by manual curation based on their ODSI values. Their preferred stimulus direction used to label the embeddings in Fig. S8a,b was computed as the argmax of the fitted tuning curves.

7. Stochastic block model

For the toy circuit examples of Fig. 6b, we ran simulations using the traditional stochastic block model (35, 36), which models a network as a partition of nodes into blocks, or clusters. Edges are established between pairs of nodes according to prespecified intra- and inter-block probabilities. Each of the three networks used 5 blocks with $n = 100$ nodes per block. The top row network was modeled using a uniform intracluster edge probability $P_{\text{intra}} = 0.1$ and intercluster probability $P_{\text{inter}} = 0.001$. The middle row network used $P_{\text{intra}} = 0.05$ and $P_{\text{inter}} = [0.005, 0.001, 0.001, 0.0001]$ as the intercluster probabilities to the other four

clusters. The bottom row network used $P_{\text{intra}} = 0.05$ and $P_{\text{inter}} = [0.025, 0.01, 0.005, 0.005]$. Adjacency matrices in Fig. 6b,c were subsampled using a maxpool operation to have comparable dimensions and facilitate visualization.

8. Deep convolutional networks

We used publicly available models of the ResNet (37) and VGG (38) network architectures, pre-trained on ImageNet (39). Both were state-of-the-art when released, excelling in tasks like classification and localization (see, e.g., 40, for a review). The former is still widely used; it introduced residual connections, a breakthrough in deep learning which has since been applied to most architectures, even to the most recent transformer networks. ResNet and VGG are particularly relevant to this study because both have been frequently used to assess how well they perform as models for the visual system in mouse (41–43) and primate (44, 45).

For their inputs (stimuli), we used cropped frames from our stimulus ensemble movie (800×600 pixels) to meet the required input size of these networks (224×224 pixels). These were fed sequentially to the networks in order to obtain temporal activity from individual neuronal units (artificial neurons). Because of the necessary cropping, all stimulus frames were uniformly rescaled to 50% of their original size in order to increase the number of visual features present in the same frame; this also helped increase the levels of activity in response to our stimuli. As in the mouse recordings, because the flow stimuli were stochastic (in position and velocity), we presented 3 different instances of each variation.

Activity levels in response to each frame were taken as the output of each unit's nonlinearity. For the classification layer of ResNet50, which uses a linear layer followed directly by a softmax operation, we half-rectified the outputs (equivalent to a ReLU). All other layers from ResNet50 (and all from VGG16) used a ReLU as nonlinearity. The numbering of layers from ResNet50 used in Figs. 5 and S9b–e follows the direct path in the network.

In analogy to what was done with the biological networks, we combined the responses to multiple trials into a single response map. Because there are no actual spikes, instead of applying the smoothing kernel (see above) we simply used a smoothing window of size 3 frames (equivalent to a 50 ms bandwidth, the maximum kernel width we used for real neurons). Receptive fields (RFs) were computed following the procedure from (46). We display RFs for units from several feature maps in Fig. 5e, uniformly scaled in size for clarity.

Sampling procedure. Each manifold was built by sampling neurons from all layers in the same stage. We first chose 40 feature maps at random, in proportion to their maximum stimulus activity, to prevent the final manifold from including, by chance, only non-responsive feature maps. To eliminate border effects, sampling was concentrated on a central region of the feature maps. 50 neurons from each map were then sampled in proportion to their maximum activity, for a total of 2000 neurons (same order of magnitude as the retina, for comparable visualization). Examples of which neuronal units were sampled from the same feature map can be seen in Fig. 5e; each receptive field (RF, see above) corresponds to a sampled unit whose position on the response map is at the center of its RF. Within the fully-connected layers, where neurons do not share weights, there is no concept of a feature map, so we selected 2000 neurons at random, again in proportion to their maximum responses.

CNNs modeling mouse visual cortex. MouseNet (47) is a recent attempt at modeling the mouse visual cortex by a deep CNN. The hyperparameters defining its architecture (e.g., filter size, connections between layers, number of channels per layer), were derived from experimental measurements from mouse visual system. The main differences from a standard CNN are the presence of parallel paths between input and output, as well as the use of a binary Gaussian mask to enforce sparseness of connections. MouseNet was trained on a classification task using a resized version of ImageNet and achieved a performance level comparable to that of VGG16.

We also analyzed AlexNet(IR) (43), which was trained with a task-agnostic, self-supervised objective function based on the concept of contrastive embeddings, also using a resized version of ImageNet. It was reported as the best predictive model of mouse visual cortical responses among a host of other architectures and objective functions, and also performed well in an ecological application.

For both networks, we used pre-trained weights available online specific to these mouse models. The analysis and data collection followed the same steps as above, except, because both were trained using low resolution images (64×64), our stimuli were rescaled accordingly. Neuronal units sampled from MouseNet were restricted to those layers explicitly modeling V1, namely VISp2/3, VISp4, and VISp5 (47). Units from AlexNet(IR) were sampled from its first two layers, since those had the highest predictivity of mouse V1 responses (43).

9. Code and data availability

Code for methods described in this paper and data used in this study are available at https://github.com/dyballa/NeuralEncodingManifolds.

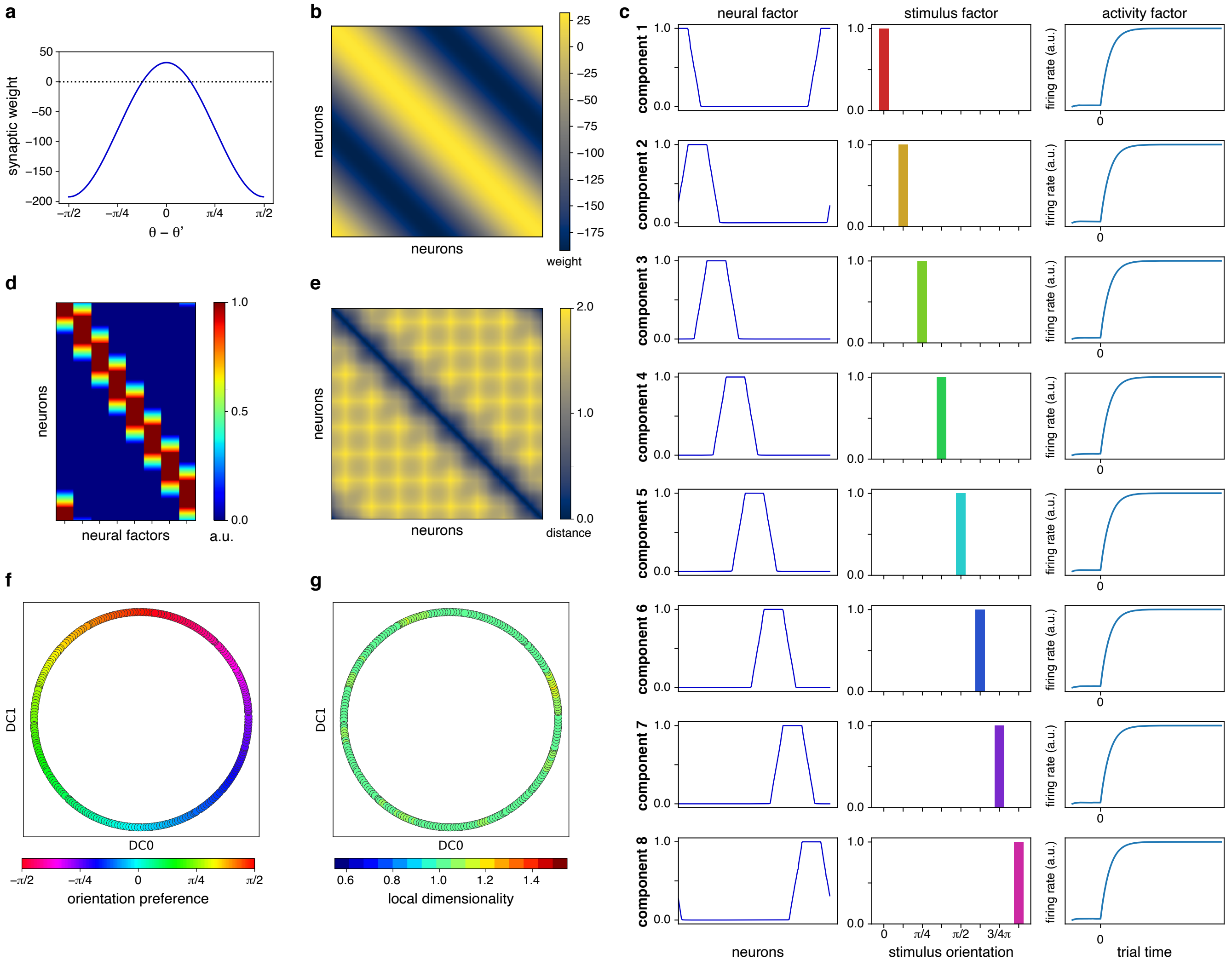

Fig. S1. Encoding manifold algorithm applied to the ring model. **a**, Synaptic connection weights between pairs of neurons indexed by difference in preferred orientation. Only similar orientations support one another; otherwise are inhibitory. **b**, Adjacency matrix representation of synaptic interactions. Columns with similar orientation preference excite and those with different preference inhibit one another (see *SI Appendix* Methods, section 2). **c**, The 8 components obtained from non-negative tensor factorization (see *SI Appendix* Methods, section 3). Response dynamics were set to be nearly identical in this simulation and appear as such. Thus the components are dictated by the stimulus (orientation) preference. **d**, Neural matrix obtained by collecting the neural factors from **c** (see *SI Appendix* Methods, section 3). Each row represents the loadings of a neuron across neural factors. **e**, The pairwise distance matrix computed from **d** and used as input to the embedding algorithm. **f**, Diffusion map embedding using the adaptive neighborhood kernel (see *SI Appendix* Methods, section 4). **g**, The estimated local dimensionality (see *SI Appendix* Methods, section 5) is 1 throughout, compatible with the single latent variable in the encoding of the stimuli used, namely orientation.

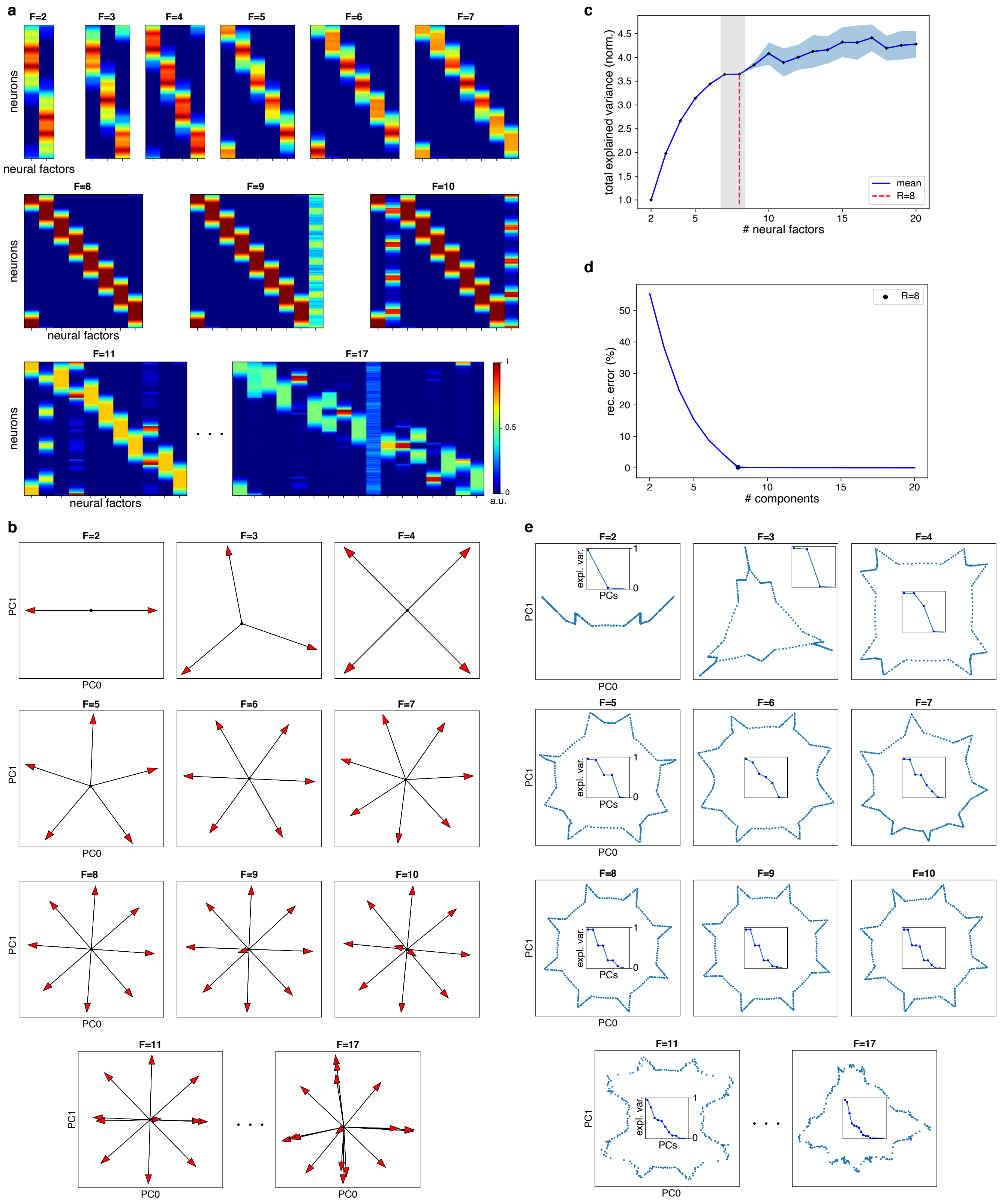

Fig. S2. *Caption on next page.*

Fig. S2. Choosing the number of tensor components, F, for the ring model.a, Neural matrices, denoted $\mathcal{N}$, resulting from various values of F. The neural loadings are most uniformly distributed when $F = 2$, 4, or 8, by symmetry, but it is only $F = 8$ that maximizes the variance explained. When $F > 8$, observe the presence of factors that either reconstruct noise (therefore explaining very little additional variance), or split previous factors into two nearly identical vectors with loadings roughly halved. Both of these situations should be avoided (see *SI Appendix* Methods, section 3). **b**, An alternate representation in which the neural factors are plotted as vectors (arrows) projected onto the first two principal components of $\mathcal{N}^T$. We now seek to maximize their span. Even though the ring can be embedded in a two-dimensional space, the "volume" defined by its neural factors finds a plateau only at F=8, since the factors are not orthogonal. This is precisely the quantity expressed by the (weighted) nuclear norm (see *SI Appendix* Methods, section 3). **c**, The total explained variance (nuclear norm) increases rapidly up to F=8, after which it flattens to a much lower rate. At this point, the variability across runs also increases markedly for larger F (cf. (4), their Fig. 2f). **d**, In this artificial example, reconstruction error alone (see Methods) is sufficient to decide the optimal choice of $F = 8$. **e**, Yet another representation: embeddings of neurons obtained by running PCA on the neural matrices from (**a**). The "ring" becomes maximally uniform at $F = 8$, and is stable until $F = 10$, above which the overfactoring makes it degenerate. Notice in particular the failure of a linear embedding algorithm to completely "unfold" the manifold (contrast these with Fig. S1f). Insets show the variance spectrum formed by the principal values.

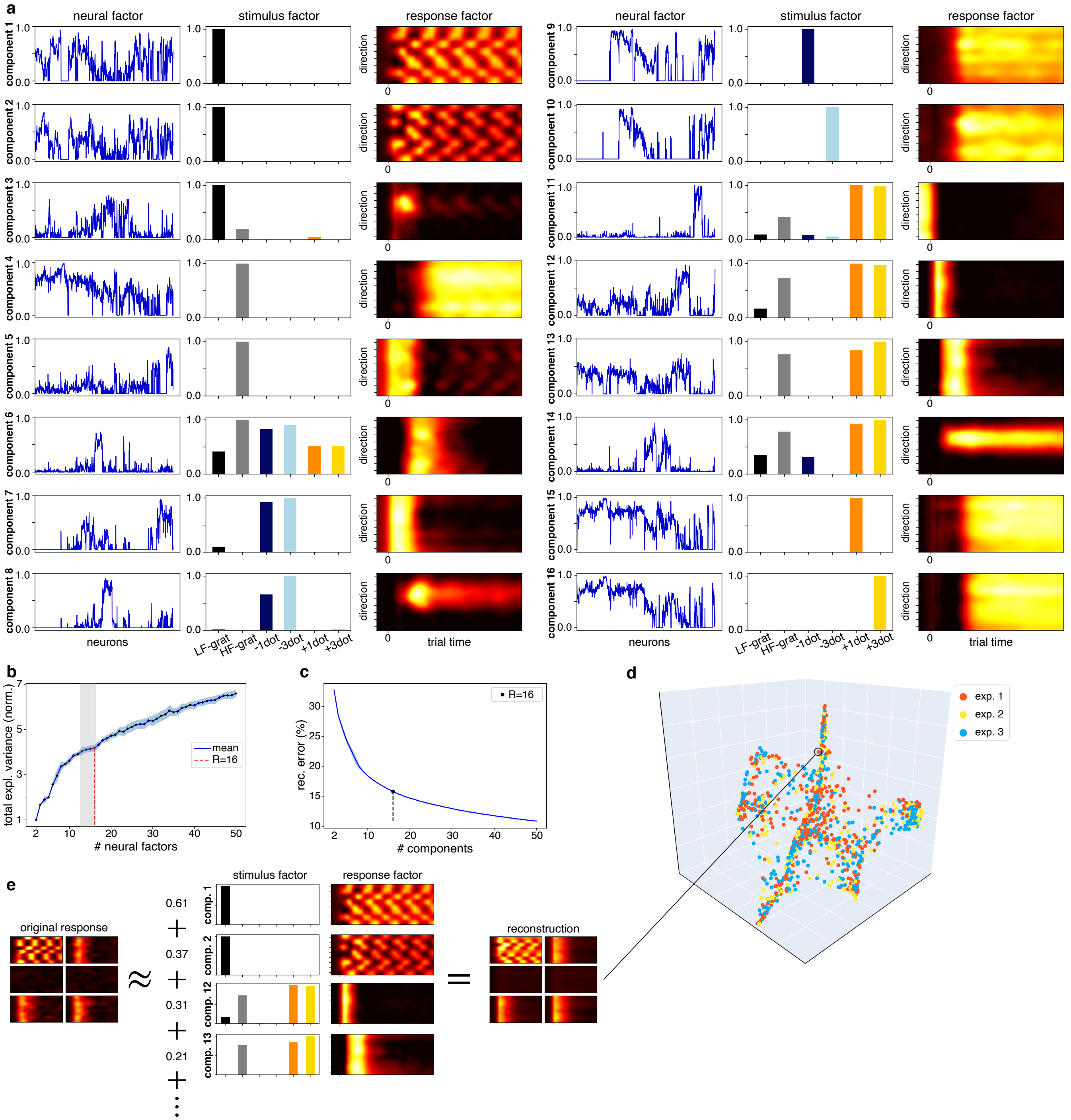

Fig. S3. Tensor decomposition for the retinal data. **a**, The three factors comprising each component. Notice how the first three components are concentrated on low spatial frequency (LF) gratings, and how other components concentrate on high frequency (HF) gratings and the various flows. Among the activity factors, the first two indicate linear-like responses, while others are either transient, sustained, tuned, or delayed. Finally, notice how the neural factors are, in general, complex mixtures. **b**, Selection of appropriate number of components ($R = 16$) based on normalized explained variance (nuclear norm, see *SI Appendix* Methods, section 3). **c**, Reconstruction error as a function of the number of components (see Methods). **d**, Multiple recordings can be combined. Shown are data from three different experiments; neurons from different retinas are well-mixed across the manifold. **e**, Example of response reconstruction for a single cell using the components from **a**. Shown are the five components with largest neural factor loadings.

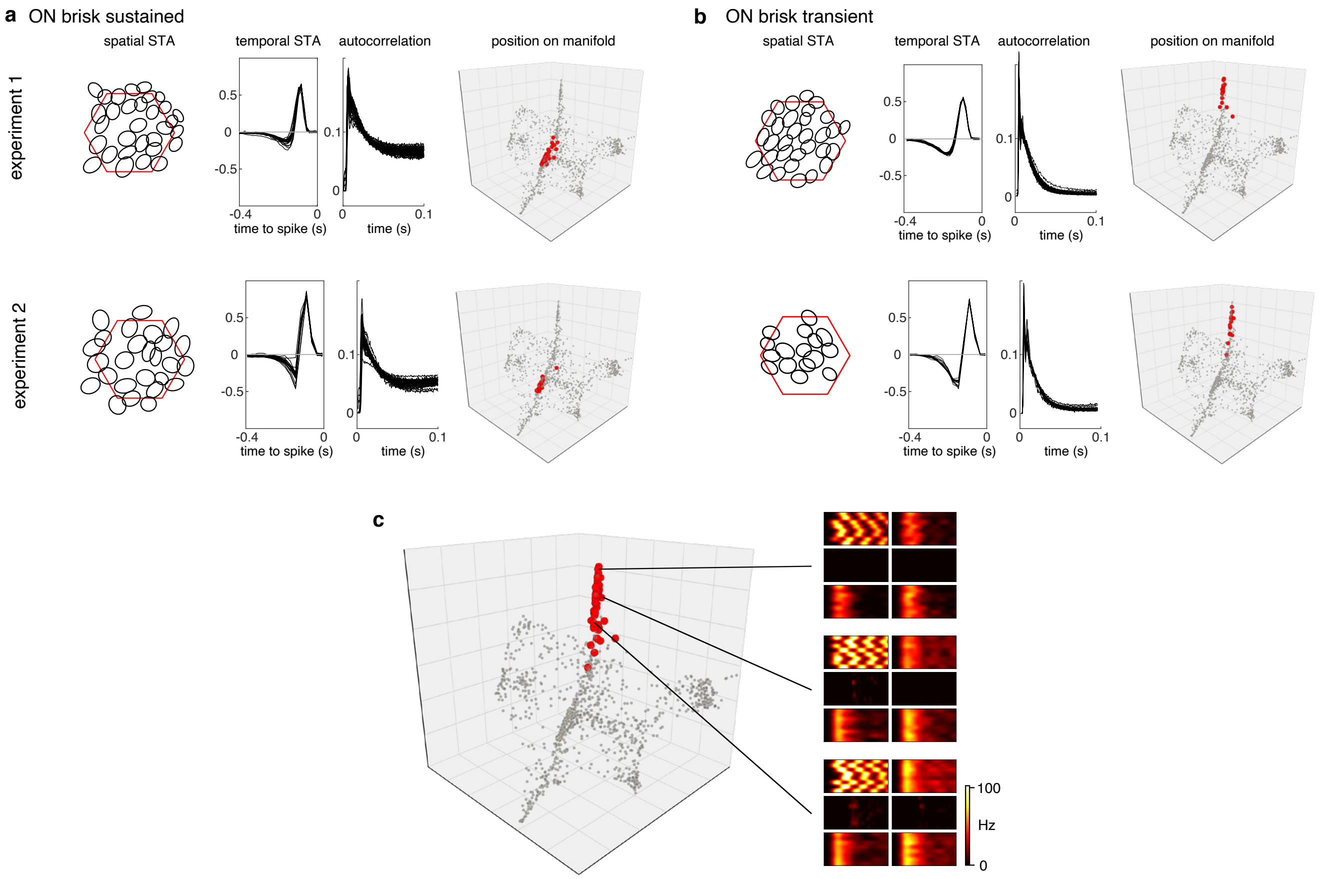

Fig. S4. Understanding the retinal encoding manifold. a–b, Examples of retinal ganglion cells (RGCs) classified as ON brisk sustained (**a**) and as ON brisk transient (**b**) (data from two experiments). The classification is based on spatiotemporal spike-triggered averages (STAs) and spike autocorrelation curves, and was done independently (used a different stimulus) from building the encoding manifold (see Material and Methods). Both of these RGC types are concentrated in specific regions (red dots) in the neural encoding manifold. **c**, ON brisk transient cells from all experiments combined in a single manifold (shown in red). Activity maps shown on the right reveal why these neurons cluster in an elongated fashion. Although qualitatively there is almost no change between them, there is a small, quantitative change in the relative magnitude of responses to high frequency gratings and positive flows compared to low frequency gratings. This slight increase, as one moves from top to bottom, is captured by the third (vertical) diffusion coordinate, highlighting an advantage of using a spectral embedding method (i.e., diffusion maps).

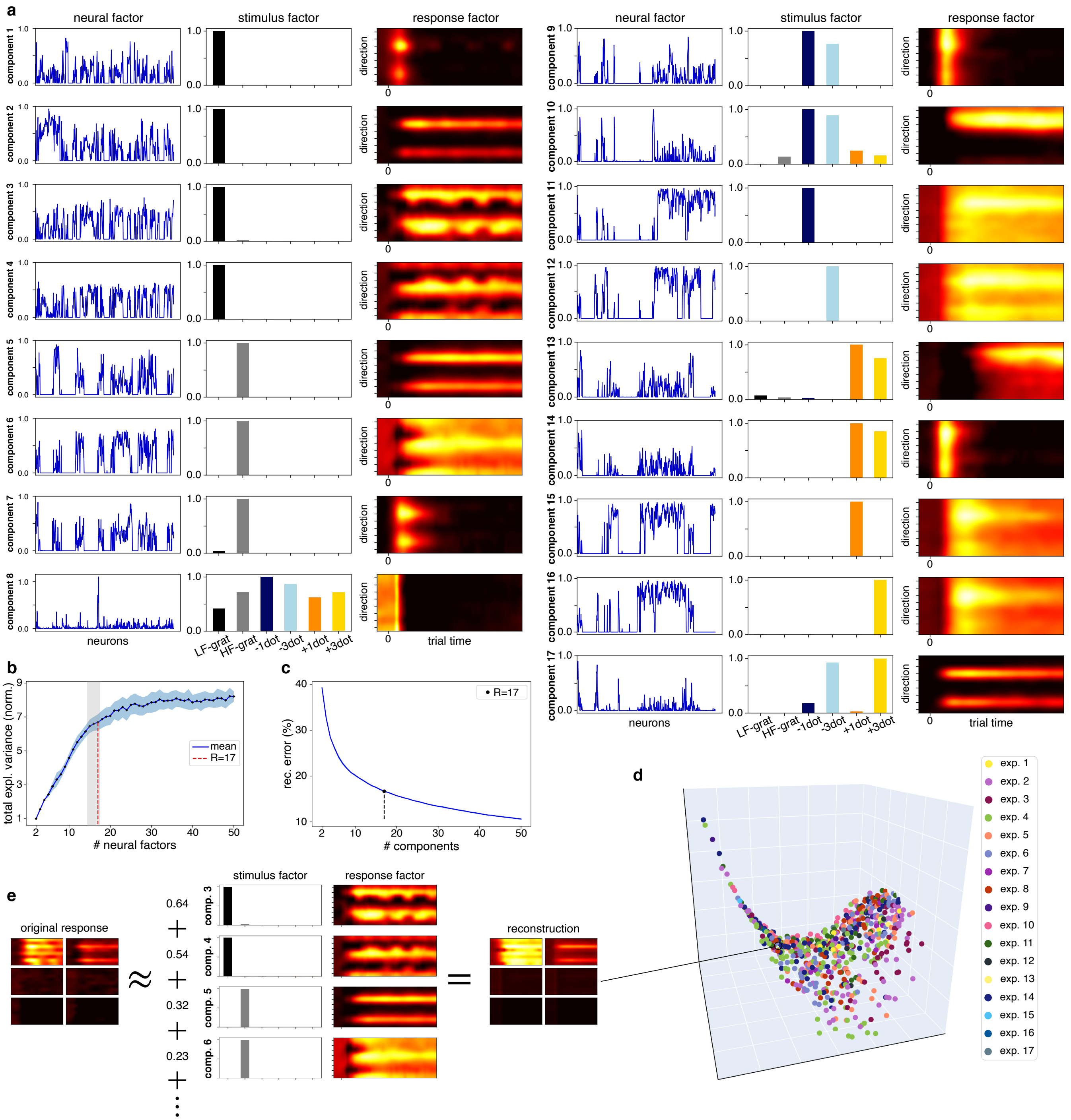

Fig. S5. Tensor decomposition for the cortical data. a, The three factors comprising each component (compare with Fig. S3). Here we find response factors with various tuning widths, direction versus orientation tuning, as well as sustained versus transient temporal activity. **b**, Selection of appropriate number of components ($R = 17$, see *SI Appendix* Methods, section 3). **c**, Reconstruction error as a function of the number of components (see Methods). **d**, Different experiments are well-mixed across the manifold. **e**, Example of response reconstruction for a single cell as a weighted sum of the stimulus $\times$ response components from **a**, with weights given by the corresponding neural loadings (top four shown).

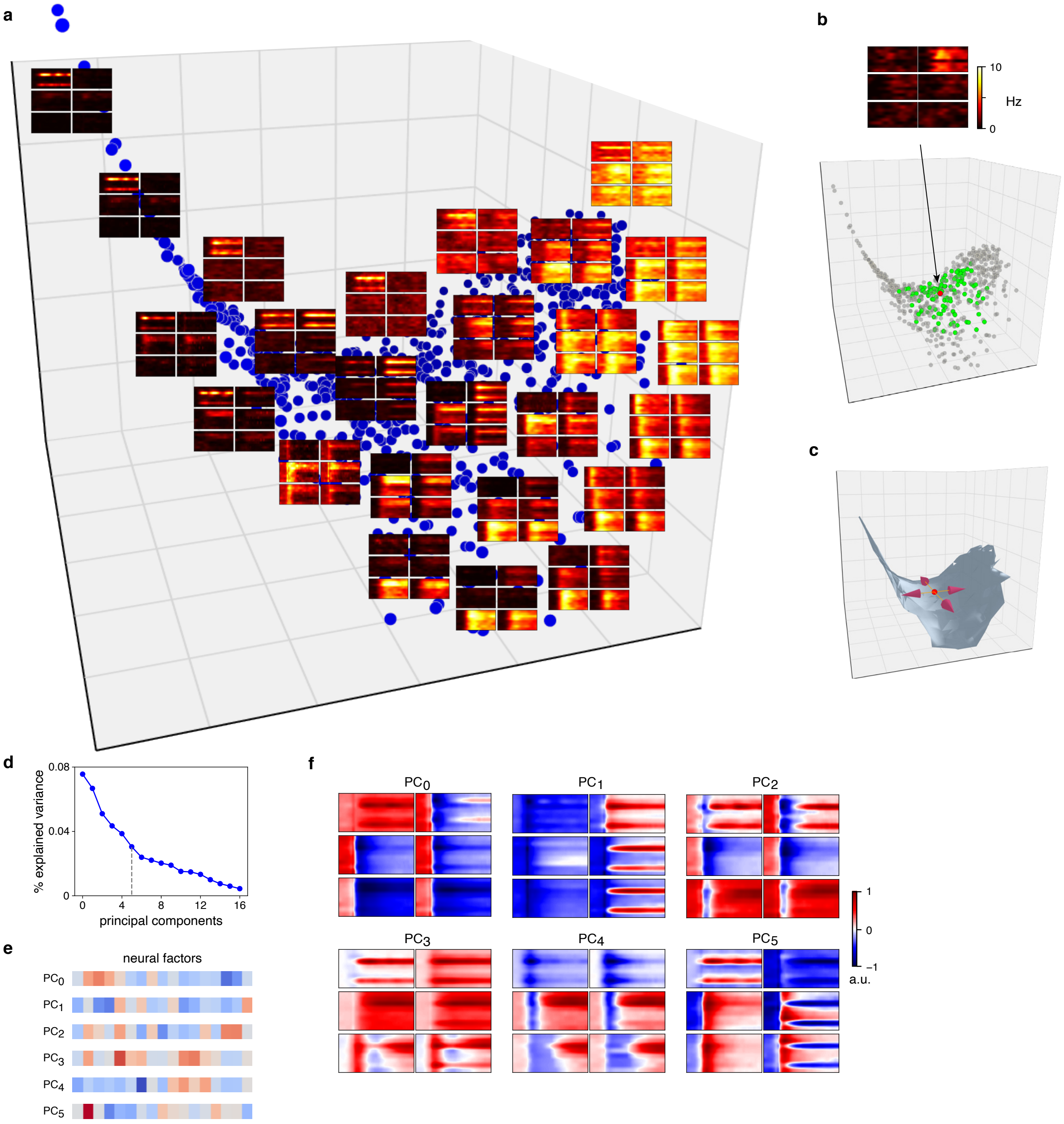

Fig. S6. *Caption on next page.*

Fig. S6. Understanding the cortical encoding manifold. **a**, Distribution of feature selectivity over the manifold. Here we show activity profile centroids (average response maps across a local neighborhood of 20 neurons) at 25 maximally distant positions (top view, as shown in inset) projected into the first three diffusion coordinates. Notice how selectivity (or visual feature preference) varies smoothly; there are no clear gaps as in the retinal manifold. The right corner shows neurons with little selectivity, and consists mostly of putative inhibitory neurons (cf. Fig. 4g). Cells responding exclusively to low-frequency gratings comprise the left arm, but responses to high-frequency gratings or flows gradually appear towards the center. The lower region is biased towards neurons responding strongly to positive contrast flows. Changing position across the manifold can be interpreted as how a neuron's stimulus/response profile changes with respect to that of another neuron. These changes can occur in more than the three dimensions that are shown here, necessitating a dimensionality analysis. **b**, Illustration of dimensionality analysis (see *SI Appendix* Methods, section 5). A neuron (ν) from the central region of the manifold, shown in red, has a local dimension of about 6 (Fig. 6), among the highest revealed by our stimuli. Nearby neurons specify a local neighborhood around ν, and have similar (but not identical) stimulus/response profiles. Mathematically, when the manifold is smooth, there is a tangent "plane" attached at ν, and moving across it indicates how the stimulus and response factors can vary as one moves a short distance along a dimension from ν. This view was discussed for the ring model in *SI Appendix* Methods, section 5, and is illustrated for V1 in **c**, showing arrows attached at ν over a continuous depiction of the cortical manifold. **d**, Performing local principal components analysis (PCA) on the neural matrix (see *SI Appendix* Methods, section 3) allows us to estimate these dimensions using ν's local neighborhood in the data graph (neurons in green). Note how the spectrum agrees with the intrinsic dimension, and **e** how this procedure allows us to express each principal component (PC), or direction, in the neural encoding space. **f**, Because each neural factor is associated with a stimulus and a response factor, each PC can be visualized as a weighted sum of those. PC0 indicates a competition (positive versus negative values) between low-frequency spatial gratings and all other stimuli, and PC1 indicates a direction in which the orientation tuning for high frequency gratings is sharpened, in concert with oriented (3-dot) flows. PC5 indicates a preference for low frequency spatial grating in concert with single dot flows, but in competition with 3-dot flows.

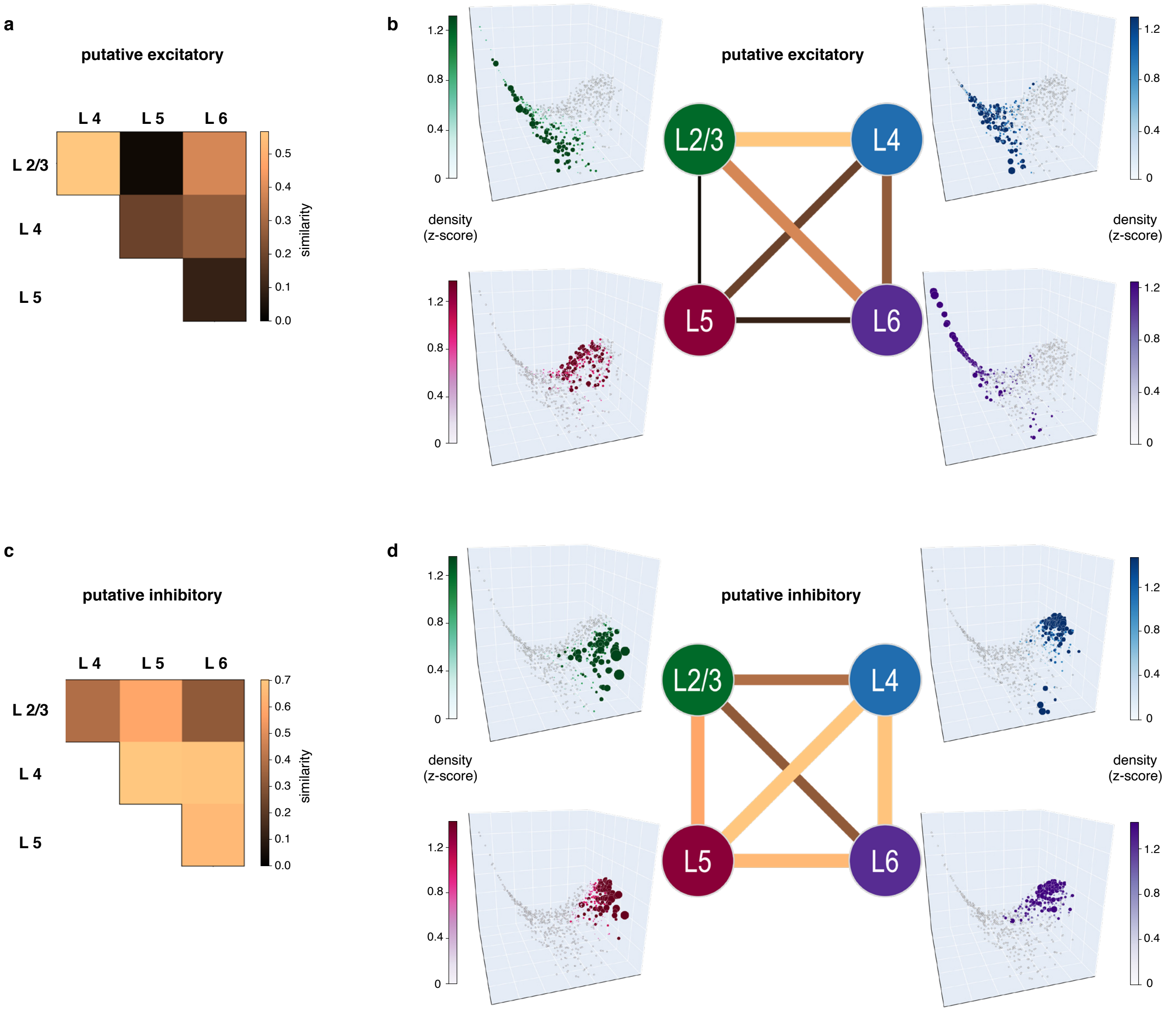

Fig. S7. Physiological implications of the V1 manifold across layers. Encoding similarity across layers in V1, computed separately for putative excitatory (**a–b**) and putative inhibitory neurons (**c–d**) (see *SI Appendix* Methods, section 4). **a,c**, Similarities visualized as a matrix. Layers 2/3 and 4 were most similar for excitatory cells, in agreement with anatomical data from (48). For inhibitory cells, layers 4, 5, and 6 were the most similar. **(b,d)** Similarities visualized as a graph in which layers are nodes and edges have thickness and color proportional to similarity, and corresponding layer densities (z-scores, see Methods) over the manifold. Because position on the manifold can be mapped back to response profiles (cf. Figs. 4 and S6), similarity between layers implies correlation between their overall feature selectivity, as indicated by responses to the stimulus ensemble. For example, for putative excitatory neurons (**b**), the high similarity between layers 2/3 and 4 follows from the common presence of neurons in both layers with narrow selectivity for gratings and/or flows (cf. Fig. S6), whereas layer 5 was the most distinct, comprised of mostly broadly selective cells (Figs. 4a,e and S6a), in agreement with data reviewed in (49). For putative inhibitory neurons (**d**), we found roughly the opposite: feature selectivity in layer 5 was highly similar to the that found in other layers, while layer 2/3 had the most distinct selectivity due to the presence of many cells responding more strongly to the flow stimuli (towards the bottom of the manifold, cf. Figs. 4a,b and S6a).

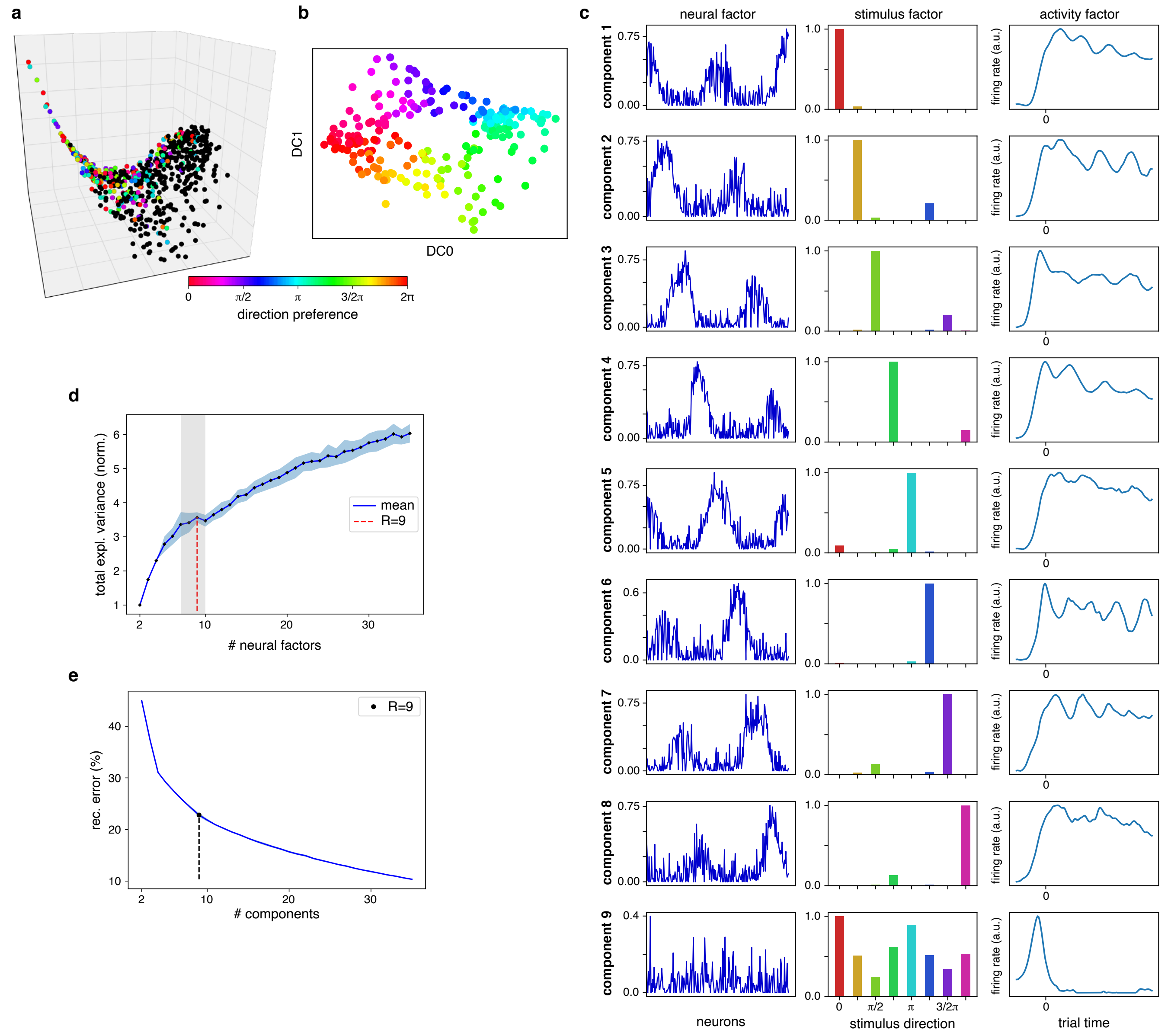

Fig. S8. Organizing cortical responses to low spatial frequency gratings. a, We here illustrate the manifold inference process without the direction of motion normalization (see Methods). The dataset is restricted to cortical cells with well-tuned responses to the orientation or direction of low-frequency gratings ($n = 225$, labeled with colors corresponding to their preferred stimulus direction), and a tensor is built where the stimulus mode is comprised of the 8 directions of motion for low frequency gratings. **b**, The result is a ring-like manifold, in analogy with Fig. S1. **c**, By our algorithm, 9 components are produced. These reveal (in contrast with those from Fig. S1c) that most of the cells have periodic response curves. **d**, Although 8 stimulus directions were used, tensor decomposition requires a 9th component (in contrast with the 8 for the artificial ring model, Fig. S2c) to account for variations in the latency period before the onset of activity. **e**, Reconstruction error as a function of the number of components (see Methods).

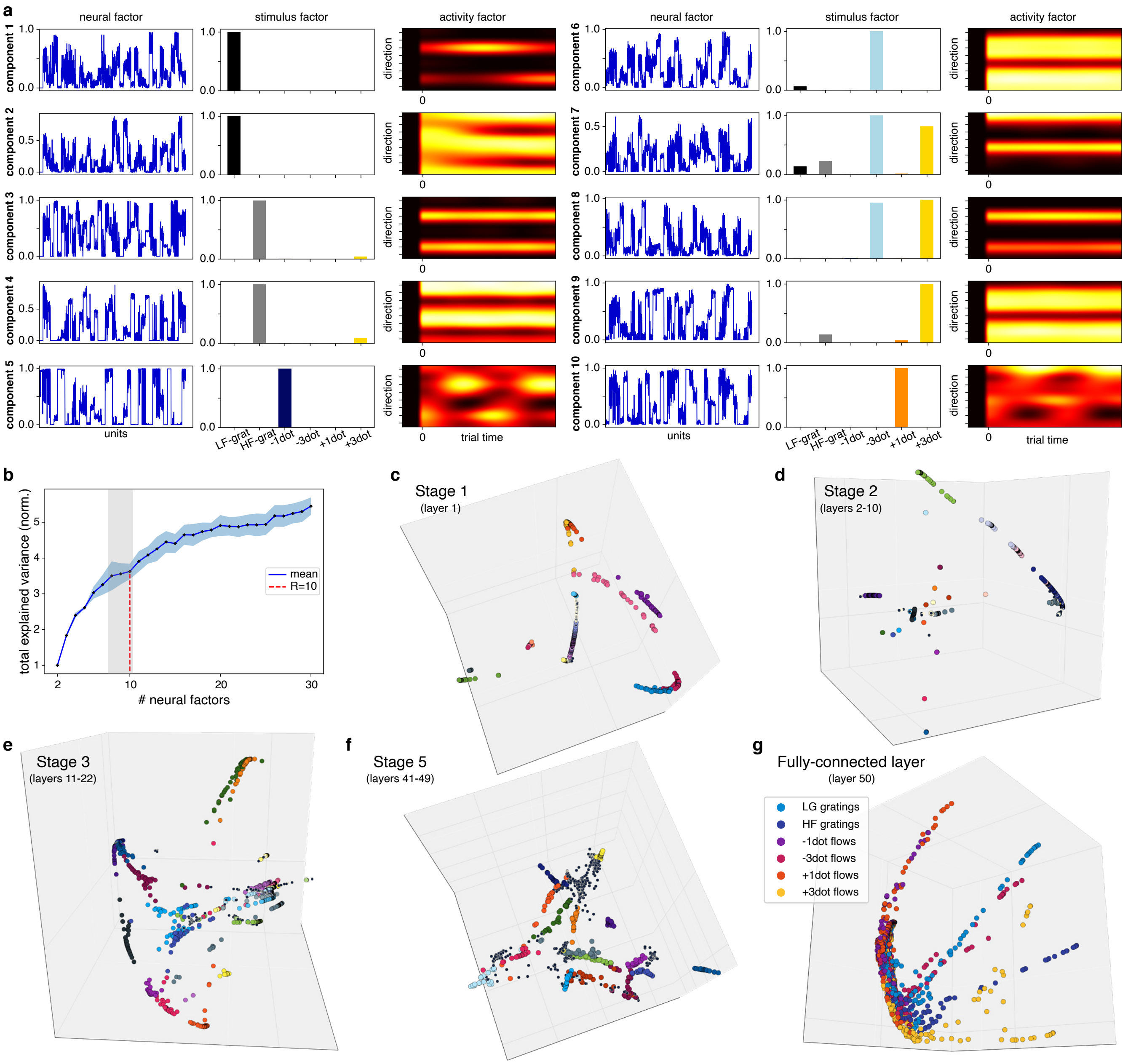

Fig. S9. Tensor decomposition for ResNet50. **a**, The three factors comprising each component (compare with Figs. S3 and S5). Notice how the stimuli organize into separate factors, and how the neural factors reflect the block structure of the network. **b**, Tensor decomposition requires only 10 factors. **c–f**, Neural encoding manifolds for the remaining convolutional stages of ResNet50 (compare with Stage 4 in Fig. 5). Color labels represent the 40 feature maps with highest activation in response to our stimuli; neuronal units were sampled at random with probability proportional to their activation (see *SI Appendix* Methods, section 8). The topology remains largely disconnected throughout the network, even when individual layers are embedded separately (as in **c**). **g**, The fully-connected layer. Because there are no feature maps, colors now represent the stimulus with highest activation for each unit. Note the specificity of stimulus preferences, which corresponds to the classification performance.

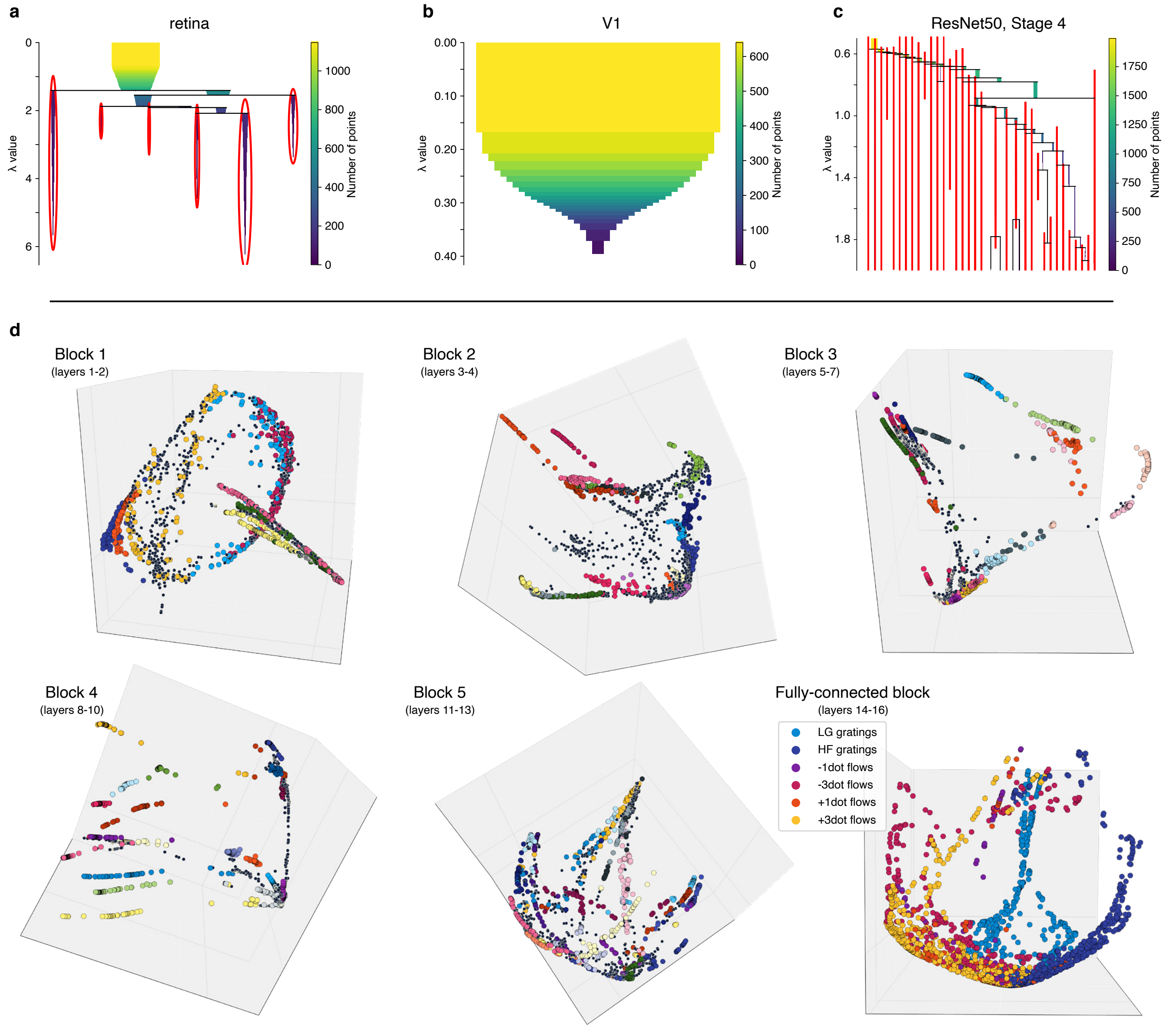

Fig. S10. Hierarchical clustering results (top) and VGG16 encoding manifolds (bottom). Using a density-based hierarchical clustering algorithm, HDBSCAN (see *SI Appendix* Methods, section 5), we inspect the resulting condensed dendrogram to assess the presence of concentrations of neurons in each manifold, i.e. how "clustered" each topology is. **a**, The retina manifold (Fig. 3), is partitioned into several clusters. **b**, For V1 (Fig. 4), the largely uniform distribution of neurons over the manifold results in an absence of clustered structure. **c**, When applied to ResNet50, stage 4 (cf. Fig. 5), a very high number of clusters are identified, matching approximately the number of sampled feature maps. **d**, Encoding manifolds for the different blocks of VGG16. The same descriptions made for ResNet50 (Fig. S9c–g) apply here.

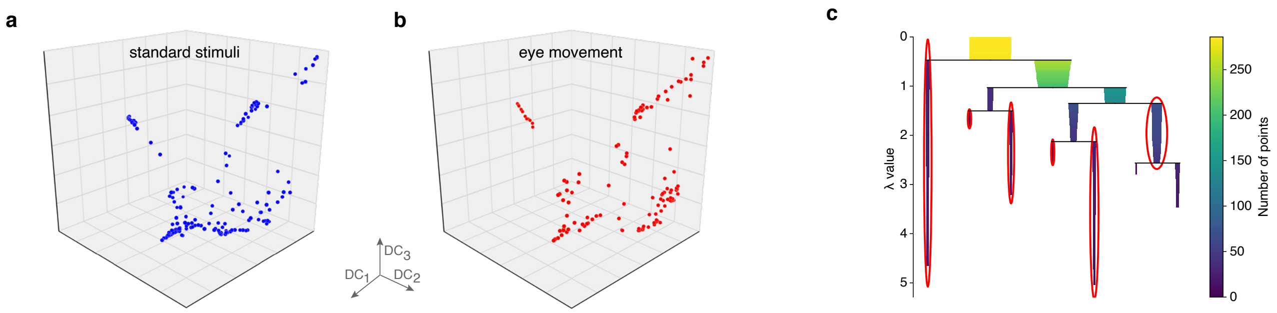

Fig. S11. Applying simulated eye movements to the stimuli has little impact on the retinal encoding manifold. An additional retinal experiment was performed in which our stimuli were translated to simulate natural eye movement data from pupil measurements of awake mice (see Methods) attending to our standard stimulus ensemble. The manifold was computed by co-embedding the neural responses from the two conditions. **a**, The manifold built from responses to the standard stimulus display ($n = 132$). **b**, The manifold built from responses to the same stimulus ensemble, modified to include the recorded eye movements ($n = 156$). Note the similarity between the manifolds. **c**, As in Fig. S10a, we used a condensed dendrogram from the HDBSCAN algorithm (see *SI Appendix* Methods, section 5) to quantify the density of clusters; note the similarity to that for our previous retinal embedding involving different retinal ganglion cells.

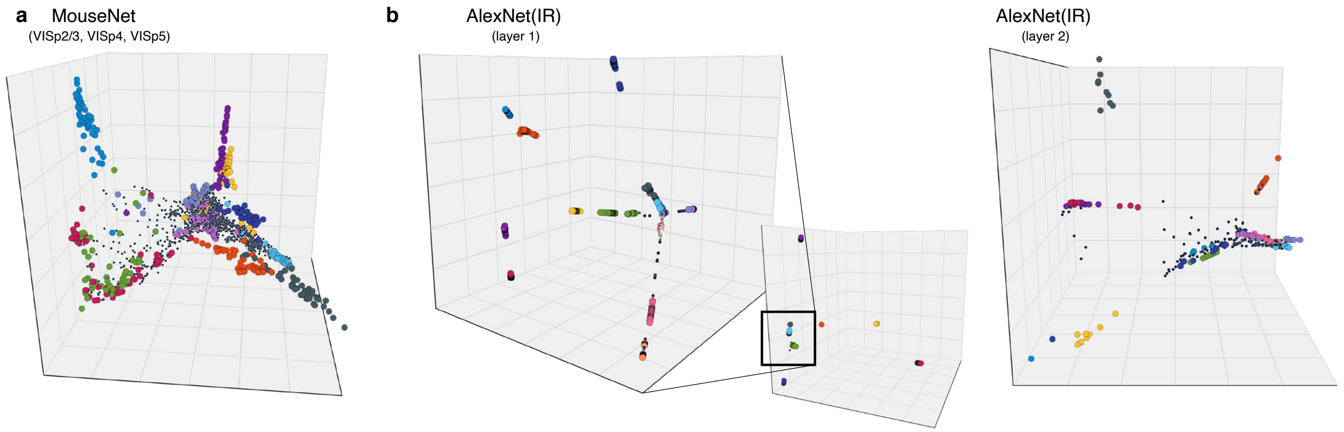

Fig. S12. Encoding manifolds for CNNs that model mouse visual cortex. a, Encoding manifold for MouseNet (47) (see Methods), including the three available layers from VISp (V1). **b**, AlexNet modified for instance recognition (IR), trained on ImageNet using a self-supervised contrastive learning scheme (43); see Methods. We here show manifolds for layers 1 (left; the zoom was necessary because of the wide diffusion distances separating the clusters) and 2 (right), which were reported to achieve the highest similarity with mouse V1 among a host of architectures (43). Note the topological similarity between these and other CNNs (Figs. S9 and S10d).

Movie S1. Animation of the retinal encoding manifold (Fig. 3) viewed from various angles, and zooming into one of the retinal ganglion cell clusters.

Movie S2. Animation of the V1 encoding manifold (Fig. 4) viewed from various angles, and showing neurons labeled by their grating selectivity index (see SI Methods).

Movie S3. Animation of the ResNet50 Stage 4 encoding manifold (Fig. 5), viewed from various angles, and zooming into a cluster of neuronal units from the same feature map.

References

1. F Pedregosa, et al., Scikit-learn: Machine learning in Python. *J Mach Learn. Res* **12**, 2825–2830 (2011).
2. R Ben-Yishai, RL Bar-Or, H Sompolinsky, Theory of orientation tuning in visual cortex. *Proc Natl Acad Sci USA* **92**, 3844–3848 (1995).
3. DH Hubel, TN Wiesel, Uniformity of monkey striate cortex: a parallel relationship between field size, scatter, and magnification factor. *J Comp Neurol* **158**, 295–305 (1974).
4. AH Williams, et al., Unsupervised discovery of demixed, low-dimensional neural dynamics across multiple timescales through tensor component analysis. *Neuron* **98**, 1099–1115 (2018).
5. JD Carroll, JJ Chang, Analysis of individual differences in multidimensional scaling via an n-way generalization of "Eckart-Young" decomposition. *Psychometrika* **35**, 283–319 (1970).
6. RA Harshman, , et al., Foundations of the PARAFAC procedure: Models and conditions for an "explanatory" multimodal factor analysis. *UCLA Work. Pap. Phonetics* **16**, 1–84 (1970).
7. TG Kolda, Multilinear operators for higher-order decompositions, (Sandia National Laboratories), Technical Report SAND2006-2081 (2006).
8. A Cichocki, R Zdunek, AH Phan, Si Amari, *Nonnegative matrix and tensor factorizations: applications to exploratory multi-way data analysis and blind source separation.* (John Wiley & Sons), (2009).
9. R Bro, S De Jong, A fast non-negativity-constrained least squares algorithm. *J Chemom* **11**, 393–401 (1997).
10. DD Lee, HS Seung, Learning the parts of objects by non-negative matrix factorization. *Nature* **401**, 788–791 (1999).
11. E Acar, DM Dunlavy, TG Kolda, A scalable optimization approach for fitting canonical tensor decompositions. *J Chemom* **25**, 67–86 (2011).
12. BW Bader, TG Kolda, Efficient MATLAB computations with sparse and factored tensors. *SIAM J Sci Comput.* **30**, 205–231 (2008).
13. TG Kolda, BW Bader, Tensor decompositions and applications. *SIAM Rev* **51**, 455–500 (2009).
14. ZI Botev, JF Grotowski, DP Kroese, Kernel density estimation via diffusion. *Ann Stat* **38**, 2916–2957 (2010).
15. T Odland, KDEpy: Kernel Density Estimation in Python (2018) v0.9.10. Available at https://doi.org/10.5281/zenodo.2392268.
16. O Christensen, *Frames and bases: An introductory course.* (Springer Science & Business Media), (2008).
17. Q Zhao, L Zhang, A Cichocki, Bayesian CP factorization of incomplete tensors with automatic rank determination. *IEEE Trans Pattern Anal Mach Intell* **37**, 1751–1763 (2015).
18. B Recht, A simpler approach to matrix completion. *J. Mach. Learn. Res.* **12** (2011).
19. L Dyballa, SW Zucker, IAN: Iterated Adaptive Neighborhoods for manifold learning and dimensionality estimation. *Neural Comput.* **35** (2023).
20. RR Coifman, et al., Geometric diffusions as a tool for harmonic analysis and structure definition of data: Diffusion maps. *Proc Natl Acad Sci USA* **102**, 7426–7431 (2005).
21. RR Coifman, S Lafon, Diffusion maps. *Appl Comput. Harmon. Anal* **21**, 5–30 (2006).
22. L Van der Maaten, G Hinton, Visualizing data using t-sne. *J Mach Learn. Res* **9** (2008).
23. L McInnes, J Healy, N Saul, L Großberger, UMAP: Uniform manifold approximation and projection. *J. Open Source Softw.* **3**, 861 (2018).
24. C Stringer, M Pachitariu, N Steinmetz, M Carandini, KD Harris, High-dimensional geometry of population responses in visual cortex. *Nature* **571**, 361–365 (2019).
25. P Gao, et al., A theory of multineuronal dimensionality, dynamics and measurement. *bioRxiv* p. 214262 (2017).
26. RJ Campello, D Moulavi, J Sander, Density-based clustering based on hierarchical density estimates in *Adv Knowl Discov Data Min: 17th Pacific-Asia Conference Proceedings, Part II 17.* (Springer Berlin Heidelberg), pp. 160–172 (2013).
27. L McInnes, J Healy, Accelerated hierarchical density based clustering in *2017 IEEE International Conference on Data Mining Workshops (ICDMW).* (IEEE), pp. 33–42 (2017).
28. K Barylyuk, et al., A comprehensive subcellular atlas of the toxoplasma proteome via hyperlopit provides spatial context for protein functions. *Cell host & microbe* **28**, 752–766 (2020).
29. M Wagner, et al., Single-cell analysis of human ovarian cortex identifies distinct cell populations but no oogonial stem cells. *Nat Commun* **11**, 1147 (2020).
30. S Avagyan, et al., Resistance to inflammation underlies enhanced fitness in clonal hematopoiesis. *Science* **374**, 768–772 (2021).

31. T Baden, et al., The functional diversity of retinal ganglion cells in the mouse. *Nature* **529**, 345–350 (2016).
32. Y Yu, JN Stirman, CR Dorsett, SL Smith, Mesoscale correlation structure with single cell resolution during visual coding. *BioRxiv* p. 469114 (2018).
33. X Han, B Vermaercke, V Bonin, Diversity of spatiotemporal coding reveals specialized visual processing streams in the mouse cortex. *Nat Commun* **13**, 3249 (2022).
34. M Mazurek, M Kager, SD Van Hooser, Robust quantification of orientation selectivity and direction selectivity. *Front Neural Circuits* **8**, 92 (2014).
35. PW Holland, KB Laskey, S Leinhardt, Stochastic blockmodels: First steps. *Soc networks* **5**, 109–137 (1983).
36. E Abbe, Community detection and stochastic block models: recent developments. *J Mach Learn. Res* **18**, 6446–6531 (2017).
37. K He, X Zhang, S Ren, J Sun, Deep residual learning for image recognition in *Proceedings of the IEEE conference on computer vision and pattern recognition.* pp. 770–778 (2016).
38. K Simonyan, A Zisserman, Very deep convolutional networks for large-scale image recognition. *arXiv preprint arXiv:1409.1556* (2014).
39. J Deng, et al., ImageNet: A large-scale hierarchical image database in *2009 IEEE Conf Comput Vis Pattern Recognit.* (IEEE), pp. 248–255 (2009).
40. L Alzubaidi, et al., Review of deep learning: Concepts, cnn architectures, challenges, applications, future directions. *J Big Data* **8**, 1–74 (2021).
41. SA Cadena, et al., How well do deep neural networks trained on object recognition characterize the mouse visual system? in *Neuro-AI Workshop at the Neural Information Processing Conference (NeurIPS).* (2019).
42. J Shi, E Shea-Brown, M Buice, Comparison against task driven artificial neural networks reveals functional properties in mouse visual cortex. *Adv Neural Inf Process. Syst* **32** (2019).
43. A Nayebi, et al., Mouse visual cortex as a limited resource system that self-learns an ecologically-general representation. *PLoS Comput. Biol* **19**, e1011506 (2023).
44. M Schrimpf, et al., Brain-score: Which artificial neural network for object recognition is most brain-like? *bioRxiv* p. 407007 (2018).
45. C Zhuang, et al., Unsupervised neural network models of the ventral visual stream. *Proc Natl Acad Sci USA* **118**, e2014196118 (2021).
46. A Araujo, W Norris, J Sim, Computing receptive fields of convolutional neural networks. *Distill* **4**, e21 (2019).
47. J Shi, B Tripp, E Shea-Brown, S Mihalas, M A. Buice, Mousenet: A biologically constrained convolutional neural network model for the mouse visual cortex. *PLoS Comput. Biol* **18**, e1010427 (2022).
48. TA Hage, et al., Synaptic connectivity to L2/3 of primary visual cortex measured by two-photon optogenetic stimulation. *eLife* **11**, e71103 (2022).
49. A Naka, H Adesnik, Inhibitory circuits in cortical layer 5. *Front. neural circuits* **10**, 35 (2016).
